# Integrated Functional Analysis of the Nuclear Proteome of Classically and Alternatively Activated Macrophages

**DOI:** 10.1155/2019/3481430

**Published:** 2019-04-30

**Authors:** John E. Wiktorowicz, Imran H. Chowdhury, Susan Stafford, Subhadip Choudhuri, Nilay Dey, Nisha J. Garg

**Affiliations:** ^1^Department of Biochemistry and Molecular Biology, University of Texas Medical Branch (UTMB), Galveston, Texas, USA; ^2^Institute for Human Infections and Immunity, UTMB, Galveston, Texas, USA; ^3^Biomolecular Resource Facility, UTMB, Galveston, Texas, USA; ^4^Department of Microbiology and Immunology, UTMB, Galveston, Texas, USA

## Abstract

Macrophages (M*φ*) play a central role in coordinating host response to pathogens, cellular injury, and environmental stimuli. Herein, we report multidimensional, nuclear proteomic analyses of protein expression and posttranslational modifications (PTMs) that control biological processes during M*φ* activation. For this, M*φ* were incubated with IFN-*γ*/LPS and IL-4, and their differentiation to proinflammatory (M1) and anti-inflammatory (M2a, referred as M2 for simplicity throughtout the manuscript) phenotypes was confirmed by detection of CD64 and CD206 surface markers and TNF-*α*, arginase I, and iNOS-dependent nitrite levels. We used a sequential method of organellar enrichment and labeling of nuclear fractions with BODIPY FL-maleimide fluorescence dye followed by two-dimensional electrophoresis (2DE) to capture quantitative changes in abundance and S-nitrosylated (SNO) proteome signatures. Exact same gels were then labeled with Pro-Q Diamond to detect protein phosphorylation. MALDI-TOF/TOF MS analysis of the protein spots with fold change of ≥|1.5| in any of the groups yielded 229 identifications. We found that 145, 78, and 173 protein spots in M1 M*φ* and 105, 81, and 164 protein spots in M2 M*φ* were changed in abundance, S-nitrosylation, and phosphorylation, respectively, with respect to M0 controls (fold change: ≥|1.5|, *p* ≤ 0.05). Targeted analysis by immunoprecipitation and Western blotting was performed to verify the differential abundance and phosphorylation levels of two of the proteins in M1 and M2 (vs. M0) M*φ*. Ingenuity Pathway Analysis of the nuclear proteome datasets showed that the abundance and posttranslational (SNO and Phosphor) modifications of the proteins predicted to be involved in cytoskeletal organization/cell movement, phagocytosis/endocytosis, and cell proliferation/cell death were differentially regulated with proinflammatory and anti-inflammatory activation of M*φ*.

## 1. Introduction

Monocytes, macrophages, and dendritic cells are the mononuclear phagocytic cells that together constitute the monocyte phagocyte system (MPS) [1]. Microenvironment stimuli determine the maturation and functional activation of monocytes and their influence as effector cells in the immune system. Traditionally, macrophage (M*φ*) activation has been described as the antigen-dependent but nonspecific microbicidal activity of M*φ* that was first observed towards BCG (bacillus Calmette-Guerin) [2]. The classical, proinflammatory activation of M*φ* by granulocyte-macrophage colony-stimulating factor (GM-CSF) and interferon gamma (IFN-*γ*) cytokine was shown to be associated with enhanced cytotoxic and antitumor properties (reviewed in [3, 4]). Subsequently, GM-CSF and interleukin 4 (IL-4) and interleukin 13 (IL-13) were shown to induce in murine M*φ* high endocytic clearance of mannosylated ligands and reduced proinflammatory cytokine secretion, referred to as an alternative phenotype different from IFN-*γ* activation [3–5]. The finding that IFN-*γ* and lipopolysaccharide (LPS) elicited inducible nitric oxide synthase- (iNOS-) dependent nitric oxide (NO), while IL-4 and IL-13 induced polyamines, led Mills et al. [6] to term classically and alternatively activated M*φ* as M1 and M2, respectively. Since then, alternatively activated M2 M*φ* are recognized to cover a continuum of functional states and are further subgrouped as M2a (induced by IL-4 and IL-13), M2b (induced by immune complexes, TLRs, and IL-1R ligands), and M2c (induced by IL-10, glucocorticoids) (reviewed in [7]). Overall, M1 M*φ* help drive the antigen-specific T helper type 1 (Th1) and T helper type 17 (Th17) cell inflammatory responses; produce proinflammatory cytokines, toxic reactive oxygen species, (ROS) and NO; and play a central role in host defense against bacterial and viral infections [3]. M2 M*φ* are suggested to drive T helper type 2 (Th2) cell response and play a central role in parasite control, wound healing, fibrosis, tumor progression, and immune regulation, though they can also cause allergic inflammation, aid the growth of tumor tissues, and can be cellular reservoirs of various pathogens [8]. Understanding the coordinated functional responses of M1 and M2 M*φ*, therefore, will be important in the pathogenesis of many types of human diseases.

The eukaryotic nucleus is a highly organized organelle that contains specific functional domains essential for normal cellular homeostasis as well as for mediating the genomic response to pathogens, cellular injury, and environmental stimuli. Specifically, because the nucleus is essential for initiating the expression of inflammatory and reparative responses in M*φ* [9, 10], organellar proteomics to characterize differential nuclear protein expression and posttranslational modifications (PTM) in proinflammatory and anti-inflammatory M*φ* is expected to provide important new information about the biology of the M*φ*.

In this study, we were interested in determining how the nuclear proteins respond to M1- and M2-polarizing stimuli by changes in abundance and posttranslational modifications and thereby shape the M*φ* response. For this, we incubated the resting M*φ* with IFN-*γ*/LPS and IL-4 for 18 h to polarize them to M1 and M2a phenotypes, respectively. We labeled the nuclear fractions with BODIPY® FL N-(2-aminoethyl) maleimide (BD), and resolved the BD-labeled nuclear fractions by two-dimensional electrophoresis (2DE). BD provides stable, specific, quantitative labeling of cysteine residues [9, 10] and allowed us to capture the global changes in abundance as well as in cysteine-S-nitrosylation (SNO) profile of proteins [11]. We chose to look at SNO posttranslational modification (PTM) because Cys-SNO is a ubiquitous, reversible posttranslational modification [12] that is shown to influence the protein-protein interactions, translocation, and subcellular localization under physiological and pathological conditions [13, 14] and may also drive the M*φ* response under various stimuli. Recognizing the duality of relationship between phosphorylation and SNO, we integrated our proteomic approach with Pro-Q Diamond (PQD) staining of the gels for the detection of phosphorylated serine, threonine, and tyrosine residues in M*φ* [15]. Changes in phosphorylation levels can also drive the protein networks associated with M*φ* response to various stimuli in health and disease. All differentially expressed protein spots were then identified by mass spectrometry. We discuss the molecular networks that utilize changes in protein abundance and posttranslational modifications (SNO and phosphorylation) in response to M*φ* polarization. Our results provide a detailed picture of the pathways by which cytokines and other stimuli may specialize the M*φ* to drive the inflammatory and anti-inflammatory immune responses and establish a novel procedure whereby phosphorylation and SNO can be measured in the same samples in the same experiment.

## 2. Materials and Methods

### 2.1. Cell Culture

The NR-9456 macrophage cells, derived from wild-type mice, were obtained from BEI Resources (NIAID, NIH). For this, murine primary bone marrow cells were immortalized by infection with the ecotropic transforming replication-deficient retrovirus J2. Characterization of these cells by BEI based on immunofluorescence, stimulation assays, and flow cytometry demonstrated that immortalized cells retain macrophage-specific morphological, functional, and surface expression properties (https://www.beiresources.org/Catalog/cellBanks/NR-9456.aspx). Numerous investigators have used NR-9456 M*φ* in studying the innate immune responses to various stimuli [16–18].

The murine M*φ* were propagated in Dulbecco's modified Eagle medium (DMEM) containing 10% irradiated fetal bovine serum (FBS), 2 mM L-glutamine, 1 mM sodium pyruvate, and penicillin-streptomycin solution (Sigma-Aldrich, St. Louis, MO). Cells were seeded in 12-well (1 × 10^6^/well) tissue culture plates, and incubated for 18 h with 100 ng/ml LPS/20 ng/ml IFN-*γ* (BioLegend, San Diego, CA) or 20 ng/ml IL-4 (Life Technologies, Carlsbad, CA) to drive proinflammatory, classically activated M1 M*φ* and anti-inflammatory, alternatively activated M2 M*φ* profiles, respectively, by following the established protocol used by us and others in peer-reviewed publications [19–21]. Macrophages incubated with media alone (referred as M0) were used as controls. All chemicals were of >99% purity and of molecular and cell biology grade.

### 2.2. Flow Cytometry

Naïve M*φ* were incubated with IFN-*γ*/LPS or IL-4 for 18 h as above, except that brefeldin A (10 *μ*g/ml, Sigma-Aldrich) was added in the final 6 h to prevent protein secretion. M*φ* (5 × 10^5^ per 100 *μ*l, 4-6 biological replicates per group) were labeled on ice for 30 min with fluorescence-conjugated APC-Cy7-anti-CD11b (M*φ* marker, 557657, BD Biosciences, San Jose, CA), BUV395-anti-F4/80 (M*φ* marker, 565614, BD Biosciences), BV711-anti-CD64 (Fc*γ*RI marker of M1 M*φ*, 139311, BioLegend), and APC-anti-CD206 (mannose receptor marker of M2 M*φ*, 17-2061-82, eBioscience, San Diego, CA) antibodies (0.5-1 *μ*g/100/*μ*l). Cells were fixed with paraformaldehyde (PFA) in permeabilization buffer (4% PFA, 0.1% saponin, 1% FBS, and 0.1% NaN_3_ in 1X PBS) and utilized for intracellular staining with FITC-anti-TNF-*α* (M1 M*φ* marker, 11-7321-82, eBioscience) and eflour-450-anti-arginase (M2 M*φ* marker, 48-3697-80, eBioscience) antibodies (0.5-1 *μ*g/100/*μ*l). Cells stained with isotype-matched IgGs were used as controls. All samples were visualized and analyzed on an LSRII Fortessa Cell Analyzer by six-color flow cytometry, acquiring 50-100,000 events in a live cell gate. Data were further analyzed by using FlowJo software (v.10.5.3; TreeStar, San Carlos, CA).

### 2.3. Nitric Oxide Release

The NO levels (an indicator of iNOS activity) in supernatants of M0, M1, and M2 M*φ* (six biological replicates per group and triplicate observations per sample) were monitored by measuring the nitrite levels, a stable nitric oxide breakdown product, by the Griess assay [22]. Briefly, 50 *μ*l of supernatant samples was incubated for 5 min with 50 *μ*l of 1% sulfanilamide/5% phosphoric acid and then with 50 *μ*l of 0.1% N-(1-napthyl) ethylenediamine dihydrochloride (Sigma-Aldrich). Formation of diazonium salt was monitored at 545 nm (standard curve, 0 to 100 *μ*M sodium nitrite).

### 2.4. Preparation of Nuclear Extracts

Macrophages were scraped from plates and washed in phosphate-buffered saline (PBS). The cell pellets were suspended in cold hypotonic buffer A (50 mM HEPES pH 7.9, 1 mM EDTA, 1 mM EGTA, 1 mM dithiothreitol (DTT), 0.1 mM phenylmethylsulfonyl fluoride (PMSF), 10 *μ*l/ml of protease inhibitor cocktail (Sigma-Aldrich), and 0.1% Igepal CA-630) containing 10 mM KCl and incubated on ice for 10 min. The lysates were centrifuged at 4000 × *g* for 30 seconds at 4°C. The supernatants were saved as cytoplasmic fractions. The pellets were resuspended in buffer B (buffer A containing 1.0 M sucrose/10 mM KCl) and centrifuged at 15,000 × *g* for 10 min at 4°C. The resultant pellets were incubated for 30 min at 4°C in buffer C (buffer A containing 10% glycerol/400 mM KCl). After incubation, samples were centrifuged at 15,000 × *g* for 10 min at 4°C, and the supernatants were stored at −80°C as nuclear fractions. Cell fractions prepared by our established fractionation protocol showed Lamin B in nuclear samples only and *β*-tubulin in cytosolic samples only, while *β*-actin was detected in all samples and offered >95% purity of nuclear and cytosolic fractions [23]. Yet, we test all nuclear fractions for the presence of Lamin B and GAPDH or *β*-tubulin levels by Western blotting, and nuclear fractions that exhibit >6% of contaminants are recentrifuged as described above to ensure purity [24].

For proteomic studies, the purified nuclei were lysed in ReadyPrep Sequential Extraction Reagent 2 (163-2103, Bio-Rad, Hercules CA) and incubated for 30 min at 22°C with 300 IU/ml of endonuclease (Sigma-Aldrich) to digest the nucleic acids. Samples were then centrifuged at 15,000 × *g* at 4°C for 30 min, and supernatants were desalted using a MicroSpin G-25 column (GE Healthcare, Piscataway, NJ) for use in proteomic profiling studies.

### 2.5. BODIPY Labeling and Two-Dimensional Electrophoresis (2DE)

BODIPY labeling and 2DE protocols are published by us elsewhere [25, 26]. Briefly, protein content in nuclear fractions of M1, M2, and M0 M*φ* (four biological replicates per group) was measured by using a Pierce Modified Lowry Protein Assay Kit (Thermo Fisher Scientific, Waltham, MA), and cysteine (cysteic acid) levels were determined by amino acid analysis (Model L8800, Hitachi High Technologies, Pleasanton, CA). One aliquot of each sample (100 *μ*g protein) was treated with 6 mM ascorbate (Asc^+^) to reduce the nitrosylated cysteine residues and make them available for dye binding, and the other aliquot (Asc^−^) was treated with 100 *μ*M neocuproine (phenanthroline derivative and chelating agent) that blocks SNO reduction and stabilizes SNO during further processing of samples [25]. A huge body of literature dating back at least ten years has established the protocols for ascorbate reduction of SNO (discussed in [27]). Even a recent paper purporting to discount the importance of SNO as an endpoint modification utilizes (and demonstrates) ascorbate's reductive selectivity [28]. All sample aliquots were dialyzed against urea buffer and labeled with BODIPY FL N-(2-aminoethyl) maleimide (Life Technologies) at 60-fold excess to cysteine residues for 2 h, and the reactions were stopped with 2-mercaptoethanol.

The BD-labeled (Asc^+^ and Asc^−^) samples (100 *μ*g protein) were separated by 2DE, employing an IPGphor multiple-sample isoelectric focusing (IEF) device (GE Healthcare) in the first dimension, and the Criterion Dodeca cell (Bio-Rad) in the second dimension, as we have described previously [26].

### 2.6. Gel Fixing, BD Imaging, PQD Staining and Imaging, and Image Processing

In total, 24 2D gels representing Asc^+^ and Asc^−^ aliquots of nuclear extracts from M0, M1, and M2 M*φ* (*n* = 4 per group) were obtained. Gels were fixed in 20% methanol/7% acetic acid/10% acetonitrile and washed with 20% ethanol/10% acetonitrile, and images for BD-labeled proteins were acquired at Ex_488nm_/Em_520/40nm_ by using a Typhoon Trio Variable Mode Imager (GE Healthcare) [29]. Since some spectral overlap can occur between BD and PQD, the gels were also scanned at Ex_532nm_/Em_560nm_ to quantify the potential BD spillover signal into the PQD window. After scanning, gels were stained for 90 min with Pro-Q Diamond (Invitrogen, Carlsbad, CA) that selectively labels the phosphoproteins in acrylamide gels, destained with 20% acetonitrile/50 mM sodium acetate (pH 4.0), and scanned with the PQD detection excitation and emission configuration to quantify the PQD fluorophore. The gain voltage was adjusted for each dye to achieve ~55,000-63,000-pixel intensity (with <6% variation) from the most intense protein spots on a gel. All PQD-stained gels were scanned at a gain voltage of 400 V.

Gel images were analyzed with SameSpots v4.6 software (TotalLab, Newcastle, U.K.). Briefly, after importing the gel images and manual and automated pixel-to-pixel alignment, the program performed automatic spot detection on all image and ensured that (1) the same number of spots were outlined on all gels and (2) the same protein was given a spot number on any gel in the experiment. Next, protein spot volumes were calculated, and normalized with respect to a “normalization reference” gel from the M0 group. We did not use the normalization algorithm provided by the SameSpots software, because the assumptions behind the program's normalization algorithm do not apply to multiplexed gels. Instead, raw spot volumes obtained from the program were used to normalize all dye set quantifications by summing the spot volume signals present within each gel and applying a bias factor to all spot volumes relative to the reference gel total spot volume. In addition, since normalization is performed to account for variable protein loading, all abundance, SNO, and PQD normalizations were performed with the sample cognate Asc^+^ BD gels.

### 2.7. Quantification of Changes in Abundance, S-Nitrosylation, and Phosphorylation Levels

The BD-labeled Asc^+^ gel images were used to obtain the quantitative data on change in protein abundance. The protein spot abundance ratios were calculated from normalized spot volumes from experimental (M1 or M2) M*φ* versus the matched spot volumes on M0 controls (Δ abundance = Asc^+^ M1 or M2/Asc^+^ M0).

To obtain quantitative data on change in SNO levels, fluorescence normalized volumes of the protein spots from BD-stained Asc^−^ gels were also calculated (Δ SNO = Asc^−^ M1 or M2 M*φ*/Asc^−^ M0 control). The ratio-of-ratio (RoR = Δ SNO/Δ abundance) values were calculated to account for changes in SNO with respect to protein abundance and establish true SNO-specific changes in experimental samples with respect to controls. Moreover, it is noted that because SNO modification prevents the Cys-BODIPY labeling, a negative value indicates an increase in SNO level (and vice versa) in the sample [30].

To obtain quantitative data on change in phosphorylation levels, the BD spillover volumes for each spot were subtracted from the corresponding PQD spot value. The corrected PQD values were then normalized for protein loading differences by using the BD normalization factor of that same gel. The differential protein phosphorylation of each spot volume was then calculated (Δ phosphorylation = PQD^+^ M1 or M2 M*φ*/PQD^+^ M0 control). Note that because the PQD gels were BD gels restained with PQD, the SNO values for a given protein spot can be directly compared to its phosphorylation state since they are determined from the exact same protein spot on the exact same gel.

The spot volumes were subjected to statistical analysis by using built-in tools of TotalLab SameSpots software. The Δ protein abundance, Δ phosphorylation, and RoR values for all protein spots between any two groups were subjected to statistical analysis by Student's *t*-tests with Welch's correction for unequal variances. Also, to account for the false discovery rate, Benjamini-Hochberg (B-H) multiple hypothesis testing correction was applied and significance was accepted at *p* value ≤ 0.05.

### 2.8. Matrix-Assisted Laser Desorption Ionization-Time of Flight (MALDI-TOF)/Mass Spectrometry (MS) for Protein Identification

The protein spots that exhibited significant differential abundance, phosphorylation, or S-nitrosylation in at least one of the sample groups (fold change: ≥|1.5|, *p* value ≤ 0.05) were subjected to mass spectrometry identification, as we have previously described [31, 32]. Briefly, 1 mm protein spots on the 2D gels were picked robotically and in-gel digested with trypsin, and peptide mixtures (1 *μ*l), were analyzed with an AB Sciex 4800 TOF/TOF 5800 Proteomics Analyzer (Sciex, Foster City, CA). The MS and MS/MS spectral data were acquired and analyzed using an Applied Biosystems software package including the 4000 Series Explorer (v.3.6 RC1) with Oracle Database Schema (v.3.19.0) and Data Version (3.80.0). The instrument was operated in a positive ion reflectron mode, with focus mass set at 1700 Da (mass range: 850–3000 Da), and MS data (1000–2000 laser shots) were acquired and averaged from each protein spot. Automatic external calibration was performed with standard peptide mixture with the reference masses of 904.468, 1296.685, 1570.677, and 2465.199 kDa. Following MALDI MS analysis, 5–10 abundant ions from each protein spot were subjected to MALDI MS/MS. A 1 kV positive ion MS/MS method was used to acquire data under postsource decay (PSD) conditions. The instrument precursor selection window was ±3 Da. Automatic external calibration was performed by using reference fragment masses 175.120, 480.257, 684.347, 1056.475, and 1441.635 (from precursor mass 1570.700) [31, 32].

The MS and MS/MS spectral data were searched against the UniProt mouse protein database (last accessed: Dec.1, 2018; 54,189 protein sequences; 20,210 predicted protein-coding genes) by using an AB Sciex GPS Explorer (v.3.6) in conjunction with MASCOT (v.2.2.07, Matrix Science, Boston MA). The MS peak filtering included the following parameters: a mass range of 800 Da to 3000 Da, minimum S/N filter = 10, and mass exclusion list tolerance = 0.5 Da, and mass exclusion list for some trypsin and keratin-containing compounds included masses (Da) 842.51, 870.45, 1045.56, 1179.60, 1277.71, 1475.79, and 2211.1. The MS/MS peak filtering included the following parameters: minimum S/N filter = 10, maximum missed cleavages = 1, fixed modification of BODIPY (C), variable modifications due to oxidation (M), precursor tolerance = 0.2 Da, MS/MS fragment tolerance = 0.3 Da, mass = monoisotopic, and peptide charges = +1. Protein spot IDs with expectation values ≤ 0.05 were considered significant. The Protein Prophet algorithm was used to assign the protein probabilities [29].

### 2.9. Principal Component Analysis

We used principal component analysis (PCA), an unsupervised, “dimension reduction” algorithm, to look for grouping among samples at the gel level, based on their similarities in the proteomic data. It calculates a linear projection of the data such that the first axis (principal component 1) shows the largest variance that could be represented by the transformation (i.e., a linear combination of translation, rotation, and scaling). The second principal component is the best direction orthogonal to the first axis that accounts for the next largest variance in the data. PCA transformed the set of original variables in the data to a set of new orthogonal variables and represented the data graphically (TotalLab Limited, http://totallab.com/home/samespots/spots-faqs/). The graphical representation permits visualization of sample sets that group close to one another.

### 2.10. Immunoprecipitation and Western Blotting

Western blotting was performed to verify the changes in abundance of two proteins. Briefly, nuclear fractions of M0, M1, and M2 M*φ* (triplicate biological replicates of each) were resolved on a 10% polyacrylamide gel by using a Mini-PROTEAN electrophoresis chamber (Bio-Rad), and proteins were transferred to PVDF membrane by using a Criterion Trans-Blot System (Bio-Rad). Membranes were blocked with 0.5% BSA in 20 mM Tris-HCl (pH 7.4)/136 mM NaCl (TBS) and 0.1% Tween 20 (TBST) for 2 h and incubated overnight at 4°C with anti-hnRNPA2/B1 (ab31645, Abcam, Cambridge, UK, 1 : 1000 dilution), anti-hnRNPA3 (25142-1-AP, Proteintech, Rosemont, IL, 1 : 500 dilution), or anti-Lamin B (Abcam, loading control, 1: 1000 dilution) antibodies. All antibodies were diluted in TBST with 0.5% BSA. Membranes were washed and incubated with HRP-conjugated secondary antibody (1 : 5000 dilutions, SouthernBiotech, Birmingham, AL), and color was developed with TMB substrate.

To verify the phosphorylation of proteins, nuclear fractions of M0, M1, and M2 macrophages were used for immunoprecipitation by using a commercially available kit (ab206996, Abcam). Briefly, nuclear protein samples (500 *μ*g) were incubated at 4°C with intermittent shaking with anti-hnRNPA2/B1 or anti-hnRNPA3 antibodies (10 *μ*g each) for 12 h and with protein A/G-Sepharose beads for 1 h. The antigen-antibody Sepharose bead complexes were precipitated by centrifugation at 2000 × *g* for 2 min and stored at -80°C. The immunoprecipitates were used for Western blotting with anti-phospho^Ser/Thr/Tyr^ (MA1-38450, Thermo Fisher Scientific) antibody at 1: 1000 dilution in TBST/5% NFDM. Membranes were then incubated with appropriate secondary HRP-conjugated antibody (1: 25,000 dilution), and color was developed as above. Images were acquired by using an ImageQuant LAS4000 system (GE Healthcare, Pittsburgh MA), and densitometry analysis of protein bands was performed by using NIH ImageJ software.

### 2.11. Ingenuity Pathway Analysis (IPA)

To assess the biological meaning of the nuclear proteome datasets, we used the IPA web-based application (Ingenuity Systems, Redwood City, CA) [33]. Briefly, the datasets for change in protein abundance, change in phosphorylation, and change in SNO levels (normalized to protein abundance) for M1, M2, and M0 M*φ* were uploaded in the IPA. The software retrieves the biological information, such as gene name, tissue-specific gene expression, function, and association with disease from the literature. Then, the datasets were integrated to define protein networks and signaling pathways that changed in abundance, SNO, or phosphorylation levels in M1 and M2 M*φ* with respect to M0 controls [26, 34].

## 3. Results

### 3.1. Polarization of M*φ* by IFN-*γ*/LPS and IL-4 Treatments

**Figure 1:**
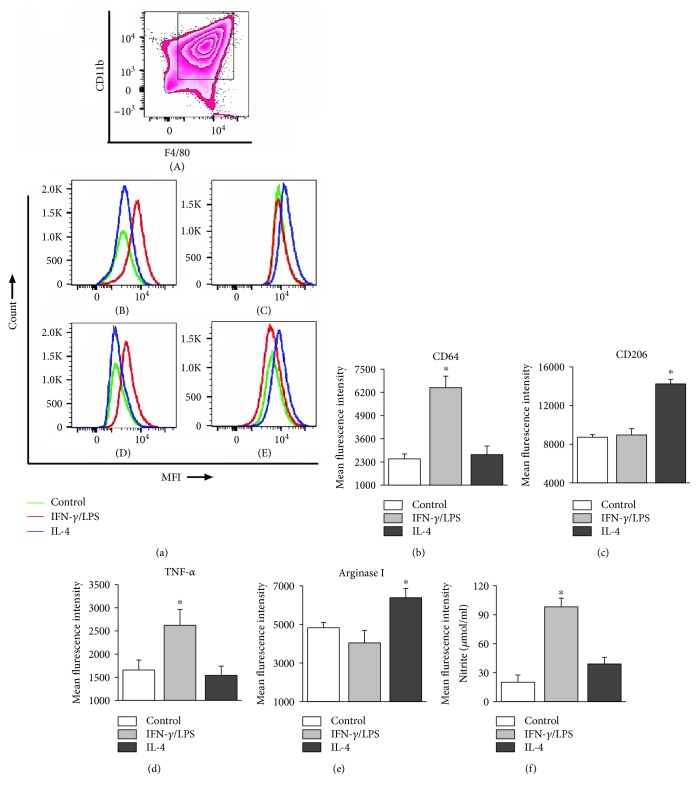
Polarization of macrophages (M*φ*). Mouse immortalized bone marrow-derived M*φ* were treated with IFN-*γ*/LPS or IL-4 for 18 h. (a) Representative flow cytometry images of the polarized subsets gated for the surface expression of CD11b and F4/80 (M*φ* markers, a) and then screened for increase in the expression of CD64 (b) and TNF-*α* (d) as the markers of classical/proinflammatory M*φ* and for CD206 (c) and arginase I (e) as the markers of alternative/anti-inflammatory M*φ* are shown. (b–e) Bar graphs show the median fluorescence intensity for the expression of CD64 (b), CD206 (c), TNF-*α* (d), and arginase I (e) in control (untreated) and IFN-*γ*/LPS- or IL-4-polarized M*φ* after 18 h of incubation (*n* = 4-6 biological replicates per group per observation). (f) Cell supernatants were used to measure the release of nitric oxide by the Griess assay (*n* = 6 biological replicates per group, triplicate observations per sample). Data in bar graphs are presented as mean value ± SD. Data were analyzed by using InStat version 3 (GraphPad, La Jolla, CA) and analyzed by the Student *t* test (for comparison of 2 groups) or one-way analysis of variance (ANOVA) with Tukey's post hoc test (for comparison of multiple groups). The significance was accepted at *p* value ≤ 0.05 (nontreated vs. treated).

We, first, assessed the efficacy of IFN-*γ*/LPS and IL-4 treatments in polarizing the M*φ* by flow cytometry. Cells were gated for CD11b^+^ and F4/80^+^ macrophage markers (Figure 1(a), A) and then screened for specific markers of M1 (proinflammatory) and M2 (anti-inflammatory) activation profile. The representative flow cytometry determination of frequencies of CD11b^+^F4/80^+^ M*φ* that expressed CD64 (Fc*γ*RI), CD206 (mannose receptor), TNF-*α*, and arginase I in IFN-*γ*/LPS- and IL-4-treated and control M*φ* are shown in Figure 1(a), B–E, and average median fluorescence intensities (MFI) are presented in Figures 1(b)–1(e). The IFN-*γ*/LPS-treated M*φ* displayed 161% and 58% increase in surface expression of CD64 (high-affinity Fc*γ*RI) and intracellular TNF-*α* levels (Figures 1(b) and 1(d), *p* < 0.05) and no increase in the expression of CD206 and arginase 1 (Figures 1(c) and 1(e)) when compared to that noted in unpolarized M*φ*. In comparison, IL-4-treated M*φ* showed 63% and 32% increase in CD206 and arginase 1 expression (Figures 1(c) and 1(e), *p* < 0.05) and no increase in the CD64 and TNF-*α* levels. The IFN-*γ*/LPS-treated M*φ* also exhibited 390% increase in nitrite release (indicates iNOS activity, Figure 1(f), *p* < 0.05), while NO was merely detectable in supernatants of IL-4-treated M*φ*. These results show that IFN-*γ*/LPS treatment led to a robust increase in the expression of phenotypic markers (CD64^+^) and functional response (TNF-*α* and NO) conducive to proinflammatory M1 M*φ* activation profile. A lack of TNF-*α* and NO production and an increase in CD206^hi^ phenotype and intracellular arginase I expression confirmed the alternative/anti-inflammatory M2 phenotype of the IL-4-treated M*φ*.

### 3.2. Schematic of Proteomic Workflow and Characteristics of Protein Spots in M1 and M2 M*φ*

**Figure 2:**
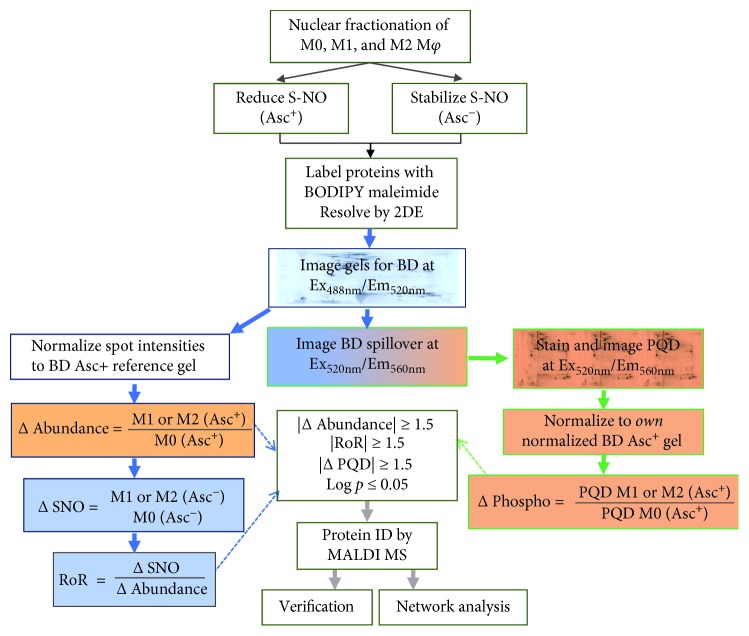
Schematic of workflow for developing the integrated proteome of M1- and M2-polarized macrophages. Mouse bone marrow M*φ* were incubated for 18 h with IFN-*γ*/LPS and IL-4 to generate M1- and M2-polarized M*φ*, respectively. Nuclear fractions were isolated as described in Materials and Methods. Each sample (*n* = 4 per group) was divided into two aliquots. One aliquot was treated with ascorbate (Asc^+^) to reduce SNO cysteines, and in the second aliquot, SNO was stabilized with neocuproine but not treated with ascorbate (Asc^−^). All aliquots were incubated with BODIPY FL N-(2-aminoethyl) maleimide (labels reduced cysteine) and resolved by two-dimensional electrophoresis (2DE). After imaging the gels for BD staining and BD spillover, each gel was stained with Pro-Q Diamond (detects phosphorylated proteins) and imaged. The differential protein abundance, SNO modification, and phosphorylation of protein spots were determined by ratiometric calculations. The fold changes in protein spots in all gels were subjected to statistical analysis, and protein spots that changed in any one of the parameter by ≥|1.5|-fold at *p* ≤ 0.05 were submitted to mass spectrometry analysis for protein identification. The proteome datasets were submitted to Ingenuity Pathway Analysis for network inquiry.

**Figure 3:**
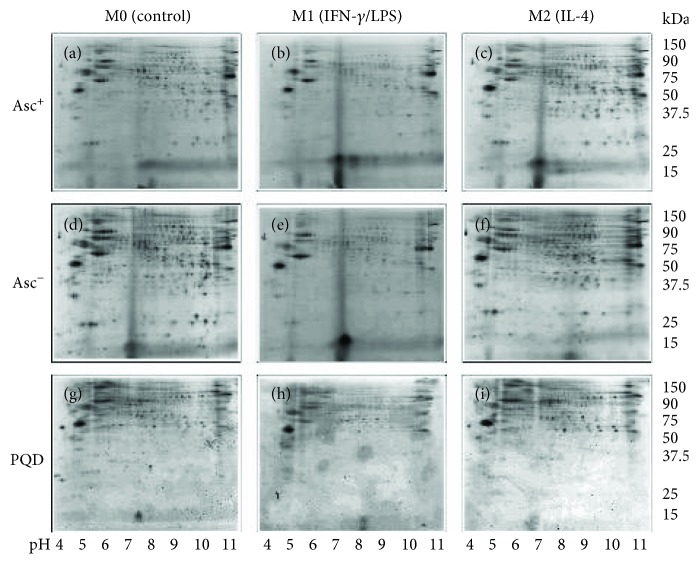
Two-dimensional gel images of protein spots in the nuclear fraction of M1, M2, and normal control M*φ*. The nuclear fractions of M0, M1, and M2 M*φ* were treated with ascorbate (Asc^+^) or neocuproine (Asc^−^) and labeled with BD. Samples were separated by 2DE (pH 3–11; 8–16% gradient gel). Gel images were obtained at 100 *μ*m resolution to quantify BD-labeled proteins (Ex_488nm_/Em_520/540nm_) and BD signal spillover into the Pro-Q Diamond window. All gels (total 24) were then stained with PQD and imaged at Ex_532nm_/Em_560nmLP_ to quantify phosphorylated protein spots. Shown are representative gel images of M0 (a, d, g), M1 (b, e, h), and M2 (c, f, i) M*φ* that were treated with ascorbate (a–c and g–i) or not treated with ascorbate (d–f) and imaged for BD labeling (a–f) and PQD (g–i) labeling. The pH values (4-11) are arrayed horizontally, and MW values (kDa) are presented vertically.

A schematic of proteomic workflow is presented in Figure 2. Briefly, each nuclear protein extract was divided into two aliquots; aliquot A was treated with ascorbate (Asc^+^) to reduce the cysteine residues, and aliquot B (Asc^−^) was treated with neocuproine to preserve SNO-modified cysteines. The BD labeling at high dye to thiol molar ratio had no effect on the isoelectric point; it was highly sensitive (detection limit: 5 fmol) and specific in quantifying the protein spots over a linear dynamic range of four orders of magnitude [35–37] and offered excellent reproducibility and accuracy. All samples were resolved by 2DE (*n* = 4 per group, total 24 gels), and BD labeling was used to measure the changes in abundance and SNO in all protein spots. Exact same gels were stained with PQD to identify and quantify those proteins containing phosphorylation. Because a small proportion (~13%) of the BD signal appears in the PQD fluorescence window, PQD signals were corrected for BD spillover signal for each spot detected (Figure 2). Representative images of gels from nuclear fractions of M0, M1, and M2 M*φ* labeled with BD (Asc^+^), BD (Asc^−^), and PQD are shown in Figures 3(a)–3(i). The spots were generally evenly distributed across both dimensions of the gel, though some protein spots appeared as horizontal “trains,” suggesting the posttranslational modifications that may alter their pIs from the parental protein. The protein spot intensities on BD-labeled and PQD-labeled Asc^+^ and Asc^−^ M1, M2, and M0 gels were normalized as described in Materials and Methods, and the data were analyzed in a pairwise manner to identify the protein spots that exhibited significant differential abundance, SNO modification, and phosphorylation at a *p* value of ≤ 0.05 in M1 or M2 M*φ* (vs. M0 controls).

**Figure 4:**
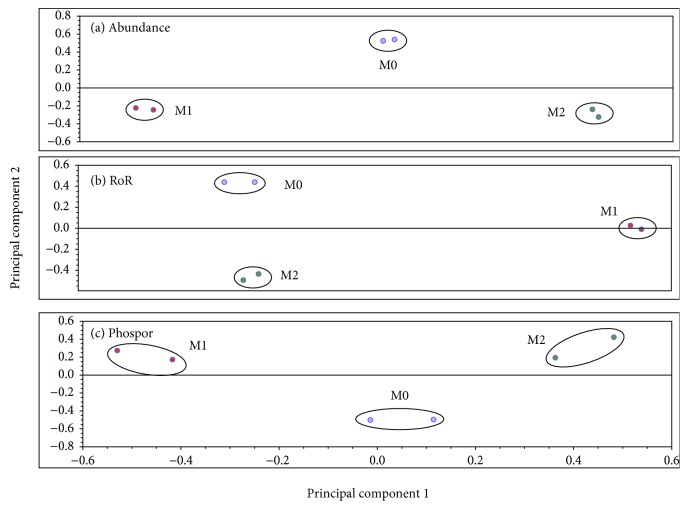
Principal component analysis. The protein spots on gels of nuclear proteins from M1, M2, and control macrophages were analyzed by PCA. (a) Protein abundance, where PC1 accounts for 52.9% of the spot variability within the gels and PC2 40.2%; (b) SNO, PC1 53.1% and PC2 40.5%; and (c) phosphorylation, PC1 42.1% and PC2 30.8%. These values confirm the impact of the M*φ* polarization on the protein abundance, SNO, and phosphorylation states.

The fluorescence signals for all protein spots on all gel images were assessed by using multifactorial principal component analysis (PCA). The PCA analysis in Figure 4 summarizes the grouping of signals from the three sets of gels. In Figure 4(a), grouping and separation of the abundance proteome profile in M1, M2, and M0 M*φ* (92.1% of the variation captured by principal components 1 (PC1) and 2 (PC2)) are apparent, with the control M0 abundance profile falling between M1 and M2 gel signals. Both M1 and M2 gel signals were widely separated from each other in PC1. In comparison, Figure 4(b)—representing signals from the SNO-labeled proteins (PC1 and PC2 accounting for 93.6% of the variability)—shows a significant difference in grouping. Within Figure 4(b), there is little difference in PC1 between the M2 and control M0 gel signals, both of which are well separated from the M1 gel signals. Figure 4(c) shows M1, M2, and M0 gels representing phosphoproteins, with PC1 and PC2 accounting for 93.6% of the variation between the three groups and M0 phosphoproteome profile falling in-between M1 and M2 gels. Overall, the PCA results demonstrated strong grouping of abundance, SNO, and phosphorylation proteome profile in M1 and M2 (vs. M0 control) M*φ*.

### 3.3. 2DE/MS Identification of Differential Nuclear Proteome in M1 and M2 M*φ*

**Figure 5:**
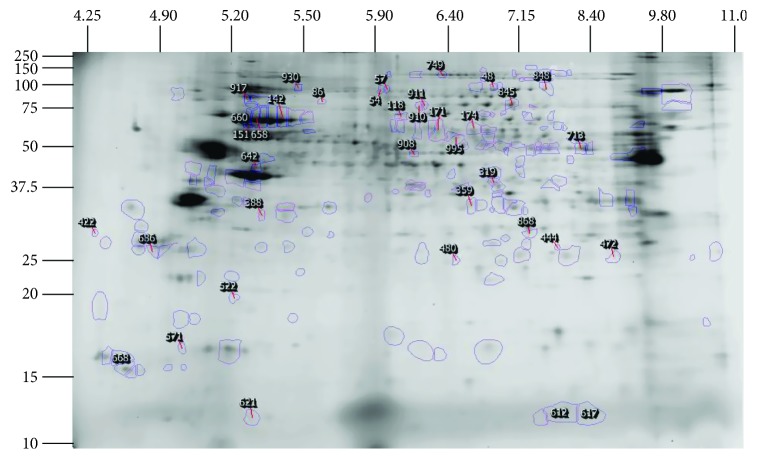
Reference gel with selected spots. Of the 995 protein spots identified on gels, 229 protein spots satisfied the selection criteria of fold change: ≥|1.5| and *p* value ≤ 0.05 as indicated in the text. The boundaries of these protein spots were identified by SameSpots software and highlighted in blue. These protein spots were robotically picked and applied for MALDI-TOF/TOF MS identification. The pI values (4-11) are arrayed horizontally, while size values (kDa) are presented vertically.

**Figure 6:**
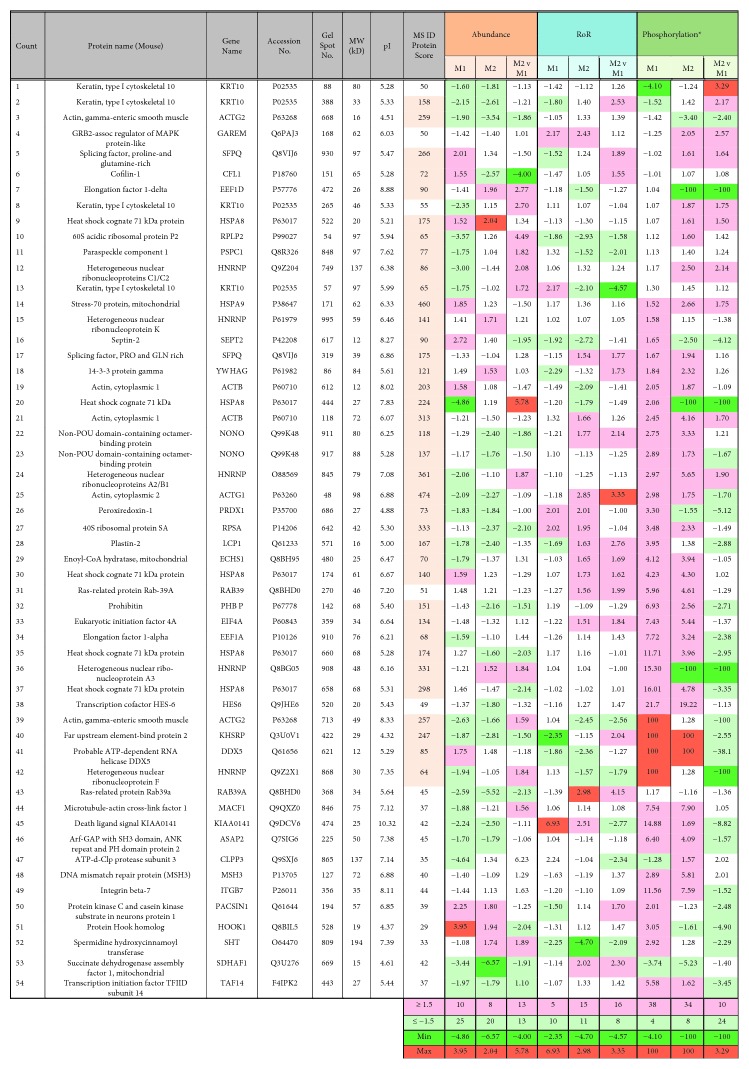
Summarized proteome profile of nuclear fractions of M1 and M2 macrophages. The nuclear protein samples from M1, M2, and M0 macrophages (*n* = 4 per group) were incubated with (Asc^+^) or without (Asc^−^) ascorbate, labeled with BODIPY FL N-(2-aminoethyl) maleimide (BD), and resolved by the 2DE approach. Gels were imaged for BD fluorescence, scanned for BD spillover in Pro-Q Diamond fluorescence detection range, and then labeled with Pro-Q Diamond and imaged for phosphorylation profile. All images were analyzed with SameSpots software; normalized spot volumes were used for comparison. Protein spots with ≥|1.5|-fold change in abundance, S-nitrosylation level, or phosphorylation level (*p* ≤ 0.05) in M1 or M2 macrophages (vs. M0 controls) were subjected to MALDI-TOF MS/MS analysis, and those identified with high confidence are highlighted in light brown color. Ratiometric calculation from BODIPY-fluorescence units in Asc^+^ aliquots (normal vs. experimental) was conducted for quantifying the differential abundance of protein spots (Δ protein abundance = Asc^+^ M1 or M2/Asc^+^ M0). The ratio of ratios, i.e., RoR = [Asc^−^ M1 or M2/Asc^−^ M0]/[Asc^+^ M1 or M2/Asc^+^ M0] was calculated to obtain the change in SNO levels normalized for protein abundance. Note that because SNO modification inhibits the Cys-BODIPY labeling, a negative RoR value indicates an increase in SNO levels (and vice versa). Ratiometric calculation from Pro-Q Diamond fluorescence units (after subtracting BD spillover) in normal vs. experimental gels was conducted for quantifying the differential phosphorylation of protein spots (Δ protein phosphorylation = M1 or M2 PQD/M0 PQD). The dark/light green and dark/light pink/orange colors indicate an increase and decrease in abundance, RoR values, and phosphorylation levels, respectively, in M1 and M2 (vs. M0) macrophages. The darker shade means the higher value.

Of the 995 protein spots detected on the reference gel, 229 protein spots exhibited significant difference (fold change: ≥|1.5|, *p* value ≤ 0.05_*t*-test/Welch/B-H_) in any one of the quantitative gel comparisons. These spots are highlighted within the reference gel in Figure 5 and were identified by MALDI-TOF/TOF analysis. The detailed proteome datasets are presented in S1 Table, and summarized proteome data focusing on protein spots identified with high confidence are presented in Figure 6. The number of protein spots that changed in any one of the parameter (fold change: ≥|1.5‐fold|, *p* value ≤ 0.05) in polarized M*φ* is presented in Table 1. Briefly, in comparison to M0 controls, several protein spots exhibited changes in abundance (M1: 37 increased/108 decreased; M2: 34 increased/71 decreased) and S-nitrosylation (M1: 36 with −ve RoR and 42 with +ve RoR; M2: 50 with −ve RoR and 31 with +ve RoR) levels in polarized M*φ*. Likewise, significant changes in the frequency of protein phosphorylation were noted in M1 (164 increased/9 decreased) and M2 (118 increased/46 decreased) M*φ*. Further, the differential abundance, SNO modification, and phosphorylation frequency of the nuclear protein spots in M1 and M2 (vs. M0 control) M*φ* ranged from 10.33-fold to −7.32-fold, 6.93-fold to −7.88-fold, and 100-fold to −100-fold, respectively (Table 1). The change in phosphorylation values (PQD^+^ M1 or M2/PQD^+^ M0) often exceeded 100-fold or −100-fold primarily because of extremely low fluorescence detected for either the M0 protein spot or the M1 or M2 protein spot. To avoid infinite fold change values in either case, we denote those as either 100- or -100-fold changes.

**Figure 7:**
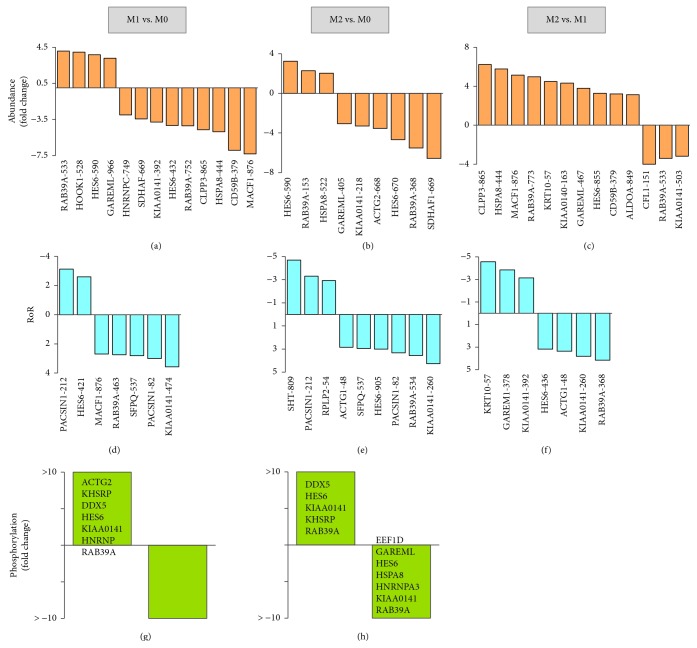
Top molecules that were differentially associated with M1 and M2 polarization of M*φ*. The bar graphs show the proteins spots that were maximally and significantly changed in abundance (a–c), SNO modification (d–f), and phosphorylation (g, h) in M1 vs. M0 M*φ* (a, d, g), M2 vs. M0 M*φ* (b, e, h), and M2 vs. M1 M*φ* (c, f). Note that a negative value for the ratio of ratio (RoR) indicates an increase in SNO modification and a positive RoR indicates decrease in SNO modification (d–f). In (g, h), data are plotted as protein spots with more than 10-fold increase or decrease in phosphorylation levels. Protein names (and spot #) are written below each category.

The top molecules that were differentially abundant, SNO-modified, and phosphorylated in nuclear fractions of M1 and M2 (vs. M0) M*φ* and in M2 (vs. M1) M*φ* are presented in Figure 7. Briefly, in M1 (vs. M0) M*φ*, the maximal increase and decrease in abundance was noted for RAB39A (FC = 4.09) and MACF1 (FC = −6.92), respectively (Figure 7(a)), while M2 (vs. M0) M*φ* exhibited maximal increase and decrease in abundance for HES6 (FC = 3.24) and SDHAF1 (FC = −6.57), respectively (Figure 7(b)). Note that the inverse in abundance of HSPA8 in M1 and M2 M*φ*s relates to different proteoforms (spot #444 of 27 kDa, pI 7.83 vs. spot #522 of 20 kDa, pI 5.21) that were reflected in M2 vs. M1 proteome signatures (Figure 7(c)). Likewise, PACSIN1 and SHT exhibited maximum increase in SNO values in M1 and M2 M*φ*, respectively (Figures 7(d) and 7(e)), and an inverse relationship in RoR values of KRT10 (spot #57) and ACTG1 (spot #48) makes these proteoform key markers of M1 and M2 phenotypes (Figure 7(f)). Several protein spots were increased by >10-fold in phosphorylation levels in M1 and M2 M*φ*, while only M2 M*φ* exhibited protein/peptides that were decreased in phosphorylation levels (vs. M0, Figures 7(g) and 7(h)). Together, these results suggest that change in abundance, SNO, and phosphorylation is a key event in regulating the M*φ* response to proinflammatory and anti-inflammatory stimuli.

### 3.4. Verification of Abundance and Phosphorylation of Proteins in Polarized M*φ*

**Figure 8:**
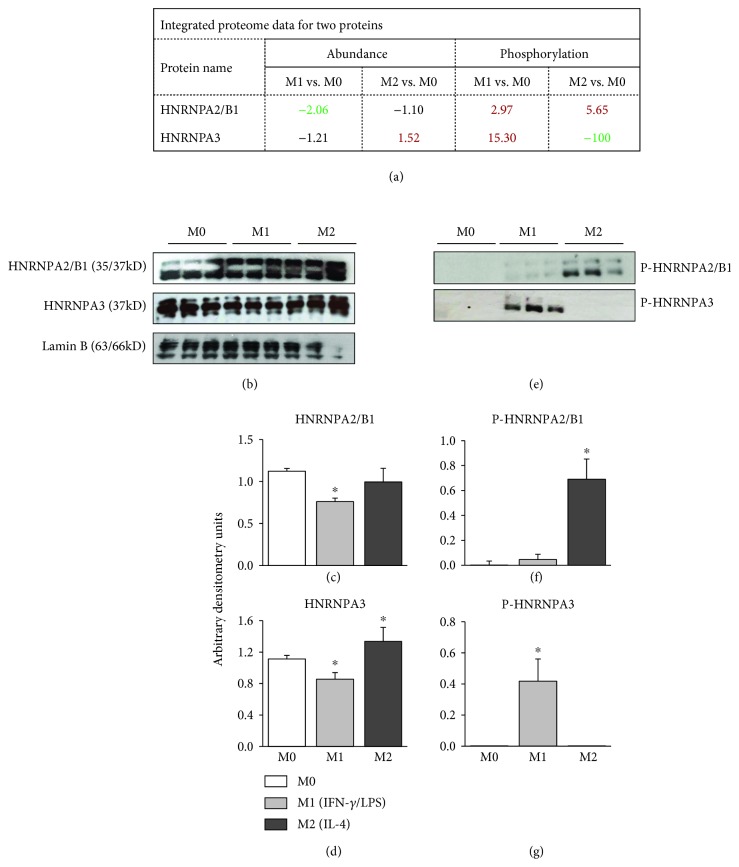
Verification of changes in abundance and phosphorylation levels of two proteins in polarized M*φ*. Macrophages were incubated for 18 h with IFN-*γ*/LPS or IL-4 to differentiate to proinflammatory (M1) and anti-inflammatory (M2) activation state, and nuclear fractions were prepared as described in Materials and Methods. (a) Proteome dataset for changes in abundance and phosphorylation of hnRNPA2/B1 and hnRNPA3. (b) Representative Western blot images are shown (3 biological replicates per group) for nuclear abundance of hnRNPA2/B1, hnRNPA3, and Lamin B (nuclear loading control) in M0, M1, and M2 M*φ*. We used 5 *μ*g, 6 *μ*g, and 12 *μ*g of each of the nuclear fractions for Western blotting for hnRNPA2/B1, hnRNPA3, and Lamin B, respectively. (c, d) Bar graphs show densitometry analyses for hnRNPA2/B1 and hnRNPA3 abundance in nuclear fractions of M*φ*, normalized to Lamin B abundance (6 biological replicates per group in two experiment sets). (e) Nuclear fractions (500 *μ*g each, three biological replicates per group) were subjected to immunoprecipitation with anti-hnRNPA2/B1 and anti-hnRNPA3 antibodies. The immunoprecipitates were resolved on 10% acrylamide gels, and Western blotting was performed with anti-phospho^Ser/Thr/Tyr^ antibody. (f, g) Densitometry analyses for phosphorylated hnRNPA2/B1 and hnRNPA3 levels, normalized to total hnRNPA2/B1 and hnRNPA3 levels, in M0, M1, and M2 M*φ* are shown. ANOVA with Tukey's post hoc test was performed to evaluate the significance presented in bar graphs (^∗^ *p* value ≤ 0.05, M1 or M2 vs. M0).

We utilized a new set of M*φ* to verify the changes in abundance and phosphorylation levels of two proteins in IFN-*γ*/LPS and IL-4 polarized M*φ* (controls, M0). We chose hnRNPA2/B1 (spot #845) and hnRNPA3 (spot #908) for these studies because these proteins, involved in packaging of nascent RNA and its cytoplasmic trafficking, may play a role in shaping the M*φ* polarization. Further, proteomic data showed that hnRNPA2/B1 was significantly decreased in abundance (M1 < M2) and increased in phosphorylation (M2 > M1), while hnRNPA3 was increased in abundance and decreased in phosphorylation in M2 M*φ* but increased in phosphorylation in M1 M*φ* (Figures 6 and 8(a)).

We first examined the abundance levels by Western blotting and found 32% and 22.5% decline in hnRNPA2/B1 AND hnRNPA3 protein levels, respectively, in M1 (vs. M0) M*φ* (*p* < 0.05, Figures 8(b)–8(d)). In M2 M*φ*, hnRNPA2/B1 was slightly, but not significantly, decreased, and the HNRNPA3 level was increased by 20% (*p* < 0.05) when compared to that noted in M0 M*φ* (Figures 8(b)–8(d)). The findings of a decline in hnRNPA2/B1 in M1 M*φ* and increase in the hnRNPA3 level in M2 M*φ* by Western blotting/densitometry were in alignment with the proteomic findings (Figures 6 and 8(a)).

For the detection of phosphorylation modification levels, we performed immunoprecipitation with protein-specific antibody, and immunoprecipitates were probed with anti-phospho^Ser/Thr/Tyr^ antibody. These data showed that the phosphorylation levels of hnRNPA2/B1 and hnRNPA3 were increased by ~4-fold and >20-fold (*p* < 0.05), respectively, in M1 (vs. M0) M*φ* (Figures 8(e)–8(g)). In M2 (vs. M0) M*φ*, hnRNPA2/B1 phosphorylation was increased by >50-fold, and hnRNPA3 phosphorylation was undetectable. Together, the results presented in Figures 8(b)–8(g) are in alignment with the findings of the changes in abundance and phosphorylation of the two proteins in the proteomic study (Figures 6 and 8(a)) and provide us confidence in the accuracy and reliability of our integrated proteomic approach in monitoring the changes in abundance and posttranslational modifications associated with M*φ* polarization.

### 3.5. Ingenuity Pathway Analysis of Integrated Nucleoproteome Associated with M*φ* Polarization

**Figure 9:**
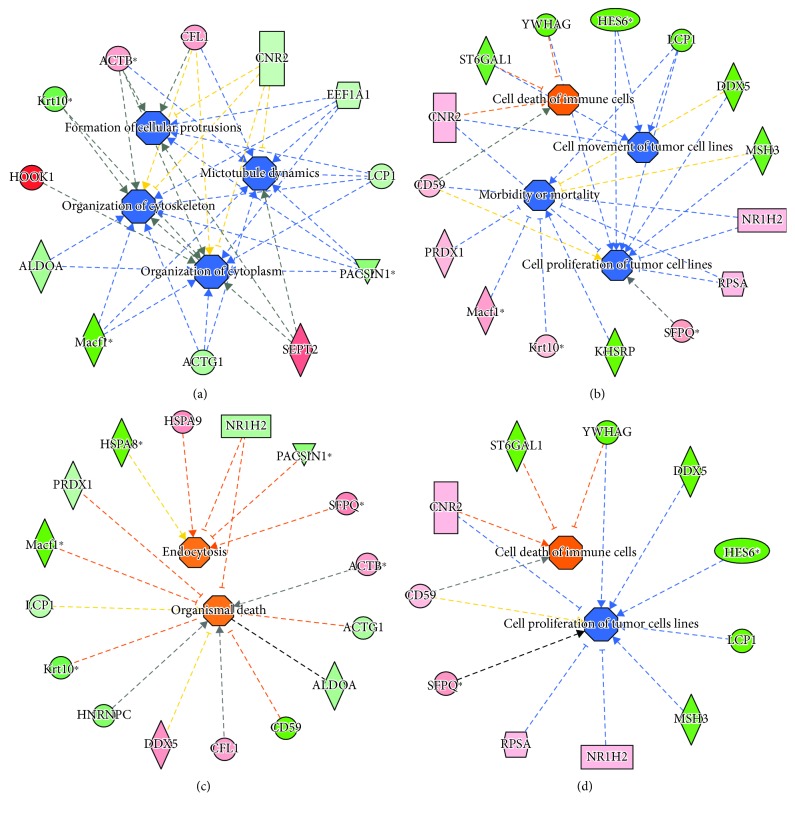
Network analysis of proteome datasets in M1 polarized (vs. M0) M*φ*. Integrated nuclear proteome of polarized and control M*φ* was developed as described in Materials and Methods, and protein spots that were differentially abundant or posttranslationally modified in M1 M*φ* (fold change: ≥|1.5|, *p* value ≤ 0.05) were identified by mass spectrometry. Ingenuity Pathway Analysis of the differential proteome datasets presented in S1 Table was performed to develop the networks. Shown are changes in abundance (a, c), RoR values indicating S-nitrosylation (b), and phosphorylation (d) of protein spots linked to disease and function networks in M1 vs. M0 M*φ*. In the networks, the intensity of pink/red and green colors shows the extent of increase and decrease in protein abundance, RoR values, or phosphorylation levels, respectively, in M1 (vs. M0) M*φ*. Brownish orange node/lines and blue node/lines show predicted activation and inhibition, respectively, of a pathway. Gray and yellow lines are used when putative effect is not completely understood. Note that several molecules that are predicted to be associated with change in cytoskeletal organization and cell movement were changed in abundance as well as S-nitrosylation, and molecules predicted to be associated with endocytosis/phagocytosis and cell death/cell survival were significantly changed in abundance and phosphorylation.

Finally, the differential proteome datasets from M1 and M2 (vs. M0) M*φ* (S1 Table) were submitted to Ingenuity Pathway Analysis to determine molecular and biological functions, as well as the important pathways and networks involved in macrophage functional activation. IPA analysis showed that a decline in abundance (Figure 9(a), *z* score: -1.525, *p* < 0.001) and increase in SNO levels (Figure 9(b), *z* score: +1.738) of several molecules were associated with inhibition of cytoskeletal/microtubule organization and cell movement in M1 M*φ*. Further, change in abundance of several molecules was predicted to be associated with increase in endocytosis/phagocytosis and cell death in M1 M*φ* (Figure 9(c), *z* score: +1.522, *p* < 0.01). Importantly, a decline in S-nitrosylation (Figure 9(b), *z* score: -2.42, *p* < 0.01) and increase or decrease in phosphorylation (Figure 9(d), *z* score: +1.732 to -1.475, *p* < 0.01) of many of the same molecules that changed in abundance were predicted to have an inhibitory effect on cell death/cell proliferation in M1 (vs. M0) M*φ*.

**Figure 10:**
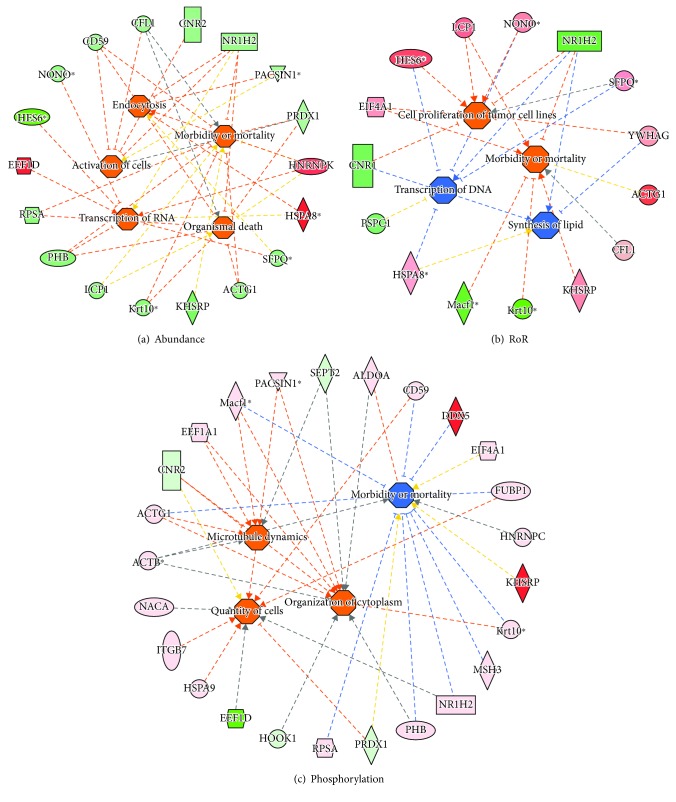
Network analysis of proteome datasets in M2 polarized (vs. M0) M*φ*. Integrated nuclear proteome of M2 (vs. M0) M*φ* was developed as described in Materials and Methods. Ingenuity Pathway Analysis of the differential proteome datasets presented in S1 Table was performed to develop the networks. Shown are changes in abundance (a), RoR values (b), and phosphorylation (c) of protein spots linked to disease and function networks in M2 vs. M0 M*φ*. In the networks, the intensity of pink/red and green colors shows the extent of increase and decrease in protein abundance, RoR values, or phosphorylation levels, respectively, in M1 (vs. M0) M*φ*. Brownish orange node/lines and blue node/lines show predicted activation and inhibition, respectively, of a pathway. Gray and yellow lines are used when putative effect is not completely understood. Note that several molecules that are predicted to be associated with increase in transcription/protein and lipid synthesis and cell proliferation and survival were changed in abundance as well as S-nitrosylation and phosphorylation levels in M2 (vs. M0) M*φ*.

IPA analysis of proteome datasets of M2 (vs. M0) M*φ* is shown in Figure 10. We noted a decline in abundance of several proteins and increase in abundance of HNRNPK, EEFID, and HSPA6 which predicted a putative increase in endocytosis (*z* score: +1.287, *p* < 0.001) and transcriptional activity (*z* score: 1.62, *p* < 0.01) associated with regulation of cell survival/cell death (*z* score: 1.336, *p* < 0.002). Importantly, S-nitrosylation of the proteins associated with regulation of cell proliferation/cell survival was decreased (*z* score: +2.61, *p* < 0.05) while others associated with transcription/translation were increased in SNO levels (*z* score: -1.587, *p* < 0.05) in M2 M*φ*. We also noted an overall increase in phosphorylation of proteins predicted to be involved in cytoskeletal/microtubule rearrangement, RNA/protein synthesis, and proliferation (*z* score: 2.122, *p* < 0.002) and inhibition of mortality (*z* score: -2.145, *p* < 0.05) in M2 (vs. M0) M*φ* (Figure 10(c)). Together, the results presented in Figures 9 and 10 suggest that changes in abundance and posttranslational (SNO and Phosphorylation) modifications of the proteins serve as an important mechanism in regulating cell proliferation, survival, and migration and in shaping the phagocytic vs. endocytic activity of the proinflammatory (M1) and anti-inflammatory (M2) M*φ*.

## 4. Discussion

S-nitrosylation and phosphorylation have emerged as key posttranslational modifications involved in regulation of physiological and pathological outcomes in cells, organelles, and tissues, particularly in neurological, cardiovascular, and oncogenic disorders (reviewed in [38–40]). More significantly, the relationship between the two modifications on protein function has been demonstrated and is critical in understanding their impacts on signal transduction and redox signaling [38, 41]. For example, SNO modification has been shown to suppress the activity of several protein kinases, including protein kinase C [42], protein kinase B [43], I*κ*B kinase [44], and calcium/calmodulin-dependent protein kinase II [45] among others through inhibiting their phosphorylation activity either by blocking the autophosphorylation or by inducing conformational changes in the kinases. SNO modification of apoptosis signal-regulating kinase-1 inhibited its binding to mitogen-activated protein kinase kinase (MKK3 and MKK6) and attenuated apoptotic cell death [46]. Likewise, SNO modification of Jun N-terminal kinase arrested its interaction with c-Jun and disrupted downstream signaling in cultured macrophages [47]. Others showed SNO modification of GRK2 (a member of the G protein-coupled receptor kinases) decreased GRK2-dependent phosphorylation of *β*-adrenergic receptor (*β*-AR) and attenuated the receptor desensitization and internalization and improved cardiac outcomes in ischemic injury [48, 49]. Recently, SNO of GSK3*β* was shown to increase the protein translocation to the nucleus where it phosphorylated nuclear substrates in myocardial cells [50]. Because several substrates of GSK3*β*, including NF*κ*B, p53, STAT3, and SIRT1, can also be modulated by SNO modification, it was proposed that SNO-GSK3*β* acts as a nuclear transnitrosylase (instead of phosphokinase) and causes SNO modification of these substrates in myocardial cells [50]. Together, these studies establish the importance of coordinated changes in posttranslational modifications, especially SNO and phosphorylation, in governing the cell-cell interactions and outcomes of the host cells (tissues) to different stimuli.

The interest in SNO and phosphorylation in inflammation and tissue repair has recently increased. Indeed, several investigators have depicted the functional role of SNO and phosphorylation of individual proteins in M*φ* activation and phenotypic outcomes [51, 52]. However, the current knowledge on the role of changes in protein abundance, SNO, and phosphorylation at the whole proteome level in response to pathological or environmental stimuli is limited. In this discovery proteomic study, we have integrated our high-throughput 2DE-MS-based approach with two protein labeling dyes (BD and PQD) for the identification and quantification of global changes in protein abundance, SNO, and phosphorylation levels in classically activated, proinflammatory (M1) and alternatively activated, anti-inflammatory (M2) M*φ* that were polarized by incubation with LPS/IFN-*γ* and IL4, respectively. The false discovery rate (*q*-value; FDR) of the protein spots satisfying abundance and *p* value boundaries was 0.026, and the SNO FDR was 0.009. Both values estimate less than one protein falsely discovered in the analysis. Importantly, we were able to establish for each protein coordinated or inverse relationship between the two PTMs. Thus, BD labeling in combination with ascorbate treatment, ability to use PQD labeling of gels, and 2DE/MS-MS offered a valuable method to identify SNO and phosphoproteomes. Of the 995 protein spots that were detected by 2DE, 229 protein spots exhibited significant differences in abundance, SNO, or phosphorylation levels (*p* < 0.05) between any of the two groups, and these were identified by mass spectrometry. Of these, five protein spots (KRT10, SEPT2, LCP1, KHSRP, and DDX5) were changed in abundance, SNO, and phosphorylation in M1 (vs. M0) M*φ* only, and four protein spots (EEF1d, NONO, ACTG1, and RPSA) were uniquely modified in abundance and PTMs in M2 M*φ* only (Figure 6 and S1 Table). We postulate that change in abundance and PTMs of these molecules would be directly involved in shaping the proinflammatory and anti-inflammatory polarization of M*φ*. If our hypothesis is proven to be correct in future studies, then these selected proteins will offer potential target molecules in clinical therapy for the infectious and chronic inflammatory diseases.

Our integrated nuclear proteome data has uncovered the main biological processes that shape the proinflammatory vs. anti-inflammatory response of the M1 and M2 polarized M*φ*. Focusing on the M1 polarized M*φ*, we observed an overall decline in abundance and phosphorylation and an increase in SNO modification of proteins involved in cytoskeleton disorganization associated with disruption of filaments that is central to formation of cellular protrusions for phagocytosis and migration in proinflammatory M1 M*φ* (Figure 9). Further, a decline in abundance and increase in phosphorylation of several proteins were associated with cell survival/cell death response (Figures 9(c) and 9(d)) in M1 M*φ*. Whether these changes in abundance and PTMs of proteins involved in immune cell proliferation, migration, and death help control the infection or diminish the cytotoxicity of inflammatory responses remains to be seen in future studies. However, we specifically discuss the proteome profile of four proteins in M1 M*φ*. Our data showed that the phosphorylation of *α*- and *γ*-actins (ACTB, ACTG1, and ACTG2) that are the highly conserved proteins of the cytoskeleton and are responsible for maintaining the cell integrity as well as are mediators of internal cell motility [53] was increased (and abundance was decreased) in M1 M*φ* (S1 Table, Figure 9). Septins are highly conserved small GTP-binding proteins that associate with actin and other microtubules and play a role in regulating membrane traffic [54]. SEPT2 is specifically found to be required for Fc*γ*R-mediated phagocytic activity in M*φ* [55]. LCP1 (also called L-plastin) is a hematopoietic-specific actin-bundling protein that is documented as both a genetic marker and a cellular mechanism contributing to invasiveness of tumors [56]. LCP1 was found to be essential for macrophage production and control of pulmonary infection in mice [57], and LCP1 null mice had a profound immune defect in controlling bacterial infections due to a lack of activation of innate immune responses [58]. In our study, the expression as well as SNO and phosphorylation levels of SEPT2 and LCP1 was increased in M1 M*φ* (Figure 9). Finally, several isoforms of Rab39a were decreased in expression and increased in phosphorylation in M1 M*φ*. Rab39a is known to have pleiotropic functions in phagosome maturation and inflammatory activation. A recent study showed that Rab39a interacts with PI3K and negatively regulates the LPS-induced autophagosome formation in M*φ* [59]. Together, our data provide strong support to the individual observations made in the literature and allow us to propose that SNO and phosphorylation posttranslational modifications of selected panel of proteins discussed here determine the phagocytic and inflammatory responses of M1 M*φ*.

In M2 M*φ*, the changes in protein expression and posttranslational modifications had a general positive regulatory effect on the molecular networks. For example, the panel of proteins that decreased in expression and SNO and increased in phosphorylation in M2 M*φ* supported the cell proliferation and survival. Specifically, the changes in abundance and SNO regulated the transcriptional activity essential for cell proliferation, and changes in abundance, SNO, and phosphorylation together influenced the cell survival, cytoskeletal remodeling, and cell motility and endocytosis functions. Focusing on some molecules of importance in shaping the molecular networks, NONO (non-POU-domain-containing octamer-binding protein) binds both DNA and RNA to regulate the expression of many genes and participates in activation of cAMP response element-binding protein target genes (e.g., TNF-*α*, IL-2, and IL-6), and it can also regulate COX2 and NF-*κ*B leading to enhanced inflammation (discussed in [60]). Our findings of a decline in abundance and SNO and increase in phosphorylation of NONO in M2 M*φ* suggest that NONO is a key regulator of anti-inflammatory phenotype of M2-like M*φ* (Figure 10). Likewise, hnRNPK, a mRNA-specific translational regulator in differentiating hematopoietic cells, was increased in M2 M*φ* (Figure 10(a)). Indeed, hnRNPK was shown to enhance the mRNA translation of TGF-*β*-activated kinase 1 (TAK1) mRNA and support TNF-*α*, IL-1*β*, and IL-10 mRNA expression [61]. Further, elongation factor EEF1D was increased in abundance and decreased in phosphorylation in M2 M*φ*. These findings suggest that a powerful stimulus of activation induced by IL-4 (vs. IFN-*γ*) alters global type of regulation, directly affecting the target molecules at the levels of both transcription and translation.

The limitations of this study are the following: firstly, we have used an established model system with a selected cell line that is of high quality and was shown to be polarized into M1 and M2 like phenotypes. However, we cannot exclude the possibility that immortalization may affect SNO and phosphorylation profiles. Studies in primary murine and human cells will be needed to confirm our findings. Secondly, we have performed all studies in M0 M*φ* that were incubated for 18 h with LPS/IFN-*γ* or IL-4 to polarize into M1 and M2 M*φ*, respectively. We recognize that our model system is established to assess the functional end points of the polarization phenotype. Similar studies at several time points during 0-18 h will be needed to determine if abundance and posttranslational modifications (SNO and phosphorylation) are dynamic and change over the course of the polarization process and to fully capture the extent of the posttranslational modifications with the development of M1 and M2 M*φ*.

In summary, we have employed a novel technology for global profiling of proteome abundance, SNO, and phosphorylation in a small amount of samples with high efficiency and specificity. Our data suggest that S-nitrosylation and phosphorylation—the two key protein modifications—govern the late-phase polarization in M*φ* conditioned by LPS/IFN-*γ* and IL-4. The phenotypic and functional categories (cell survival and proliferation/cell death, cell movement, and phagocytosis/endocytosis) were of particular significance because they appear to form the basis of selective immune polarization of M*φ*.

## Figures and Tables

**Table 1:** Summary of 2DE/MS results from nuclear fractions of M1 and M2 macrophages.

Proteomics profile	Fold change	M1 vs. M0		M2 vs. M0		M2 versus M1	
		Number	Max/Min	Number	Max/Min	Number	Max/Min
Increased abundance	≥1.5	37	10.33	34	3.24	89	6.23
Decreased abundance	≥-1.5	108	-7.32	71	-3.63	50	-6.84
Increased SNO (-ve RoR)	≥-1.5	42	-7.88	31	-4.70	38	-4.57
Decreased SNO (+ve RoR)	≥1.5	36	6.93	50	4.27	66	5.73
Increased phosphorylation	≥1.5	164	6.93	118	100	40	3.90
Decreased phosphorylation	≥-1.5	9	-7.88	46	-100	122	-100

Shown are the numbers of protein spots that exhibited protein differential abundance, SNO modification, or phosphorylation in nuclei of M1 vs. M0, M2 vs. M0, and M2 vs. M1 macrophages (fold change: ≥|1.5|, *p* < 0.05_*t*−test/Welch/B−H_). These protein spots were subjected to MALDI-TOF/TOF MS for protein identification, and complete proteome datasets are presented in S1 Table.

## Data Availability

The data used to support the findings of this study are included within the article.

## Supplementary Materials

Supplementary Materials The bone-marrow derived murine macrophages were differentiated to proinflammatory (M1) and immunomodulatory (M2) phenotype by incubation with LPS/IFN-gamma and IL-4, respectively. Samples were incubated with (Asc^+^) or without (Asc^−^) ascorbate, resolved by the 2DE approach. Gels were labeled with BODIPY® FL N-(2-aminoethyl) maleimide and ProQ, images were analyzed with SameSpotst™ software, and normalized spot volumes were used for comparison. Protein spots with ≥|1.5|-fold change in abundance, S-nitrosylation level, or phosphorylation level (*p* < 0.05) in M1 or M2 macrophages (vs. control) were subjected to MALDI-TOF MS/MS analysis. pI: isoelectric pH; MW: molecular weight.

## References

[1] Gordon S. (2003). Alternative activation of macrophages. *Nature Reviews Immunology*.

[2] Mackaness G. B. (1968). The immunology of antituberculous immunity. *The American Review of Respiratory Disease*.

[3] Shapouri-Moghaddam A., Mohammadian S., Vazini H. (2018). Macrophage plasticity, polarization, and function in health and disease. *Journal of Cellular Physiology*.

[4] Li C., Xu M. M., Wang K., Adler A. J., Vella A. T., Zhou B. (2018). Macrophage polarization and meta-inflammation. *Translational Research*.

[5] Cassetta L., Cassol E., Poli G. (2011). Macrophage polarization in health and disease. *Scientific World Journal*.

[6] Mills C. D., Kincaid K., Alt J. M., Heilman M. J., Hill A. M. (2000). M-1/M-2 macrophages and the Th1/Th2 paradigm. *Journal of Immunology*.

[7] Martinez F. O., Gordon S. (2014). The M1 and M2 paradigm of macrophage activation: time for reassessment. *F1000Prime Reports*.

[8] Wynn T. A., Chawla A., Pollard J. W. (2013). Macrophage biology in development, homeostasis and disease. *Nature*.

[9] Pourcet B., Pineda-Torra I. (2013). Transcriptional regulation of macrophage arginase 1 expression and its role in atherosclerosis. *Trends in Cardiovascular Medicine*.

[10] Lam M. T. Y., Cho H., Lesch H. P. (2013). Rev-Erbs repress macrophage gene expression by inhibiting enhancer-directed transcription. *Nature*.

[11] Miseta A., Csutora P. (2000). Relationship between the occurrence of cysteine in proteins and the complexity of organisms. *Molecular Biology and Evolution*.

[12] Htet Hlaing K., Clement M. V. (2014). Formation of protein S-nitrosylation by reactive oxygen species. *Free Radical Research*.

[13] Gould N., Doulias P. T., Tenopoulou M., Raju K., Ischiropoulos H. (2013). Regulation of protein function and signaling by reversible cysteine S-nitrosylation. *The Journal of Biological Chemistry*.

[14] Sun J., Steenbergen C., Murphy E. (2006). S-nitrosylation: NO-related redox signaling to protect against oxidative stress. *Antioxidants & Redox Signaling*.

[15] Agrawal G. K., Thelen J. J. (2009). A high-resolution two dimensional gel- and Pro-Q DPS-based proteomics workflow for phosphoprotein identification and quantitative profiling. *Methods in Molecular Biology*.

[16] Bussey K. A., Reimer E., Todt H. (2014). The gammaherpesviruses Kaposi’s sarcoma-associated herpesvirus and murine gammaherpesvirus 68 modulate the Toll-like receptor-induced proinflammatory cytokine response. *Journal of Virology*.

[17] Cuevas C. D., Ross S. R. (2014). Toll-like receptor 2-mediated innate immune responses against Junín virus in mice lead to antiviral adaptive immune responses during systemic infection and do not affect viral replication in the brain. *Journal of Virology*.

[18] Cuevas C. D., Lavanya M., Wang E., Ross S. R. (2011). Junin virus infects mouse cells and induces innate immune responses. *Journal of Virology*.

[19] Huante M. B., Gupta S., Calderon V. C. (2016). Differential inflammasome activation signatures following intracellular infection of human macrophages with *Mycobacterium bovis* BCG or *Trypanosoma cruzi*. *Tuberculosis*.

[20] Stein M., Keshav S., Harris N., Gordon S. (1992). Interleukin 4 potently enhances murine macrophage mannose receptor activity: a marker of alternative immunologic macrophage activation. *The Journal of Experimental Medicine*.

[21] Sysoliatin N. G., Ishchenko N. A., Zheleznyi P. A., Golod B. B. (1977). Surgical treatment of fractures of the mandibular articular process accompanied by dislocation of the articular head. *Stomatologiia*.

[22] Kleinbongard P., Rassaf T., Dejam A., Kerber S., Kelm M. (2002). Griess method for nitrite measurement of aqueous and protein-containing samples. *Methods in Enzymology*.

[23] Jamaluddin M., Wiktorowicz J. E., Soman K. V. (2010). Role of peroxiredoxin 1 and peroxiredoxin 4 in protection of respiratory syncytial virus-induced cysteinyl oxidation of nuclear cytoskeletal proteins. *Journal of Virology*.

[24] Wen J. J., Yin Y. W., Garg N. J. (2018). PARP1 depletion improves mitochondrial and heart function in Chagas disease: effects on POLG dependent mtDNA maintenance. *PLoS Pathogens*.

[25] Guo J., Gaffrey M. J., Su D. (2014). Resin-assisted enrichment of thiols as a general strategy for proteomic profiling of cysteine-based reversible modifications. *Nature Protocols*.

[26] Koo S., Spratt H. M., Soman K. V. (2016). S-Nitrosylation proteome profile of peripheral blood mononuclear cells in human heart failure. *International Journal of Proteomics*.

[27] Derakhshan B., Wille P. C., Gross S. S. (2007). Unbiased identification of cysteine S-nitrosylation sites on proteins. *Nature Protocols*.

[28] Wolhuter K., Whitwell H. J., Switzer C. H., Burgoyne J. R., Timms J. F., Eaton P. (2018). Evidence against stable protein S-nitrosylation as a widespread mechanism of post-translational regulation. *Molecular Cell*.

[29] Zago M. P., Wiktorowicz J. E., Spratt H. (2018). Potential utility of protein targets of cysteine-s-nitrosylation in identifying clinical disease status in human Chagas disease. *Frontiers in Microbiology*.

[30] Wiktorowicz J. E., Stafford S. J., Garg N. J. (2017). Protein cysteinyl-s-nitrosylation: analysis and quantification. *Methods in Enzymology*.

[31] Dhiman M., Zago M. P., Nunez S. (2012). Cardiac oxidized antigens are targets of immune recognition by antibodies and potential molecular determinants in Chagas disease pathogenesis. *PLoS One*.

[32] Wen J.-J., Zago M. P., Nuñez S., Gupta S., Burgos F. N., Garg N. J. (2012). Serum proteomic signature of human chagasic patients for the identification of novel potential protein biomarkers of disease. *Molecular & Cellular Proteomics*.

[33] Thomas S., Bonchev D. (2010). A survey of current software for network analysis in molecular biology. *Human Genomics*.

[34] Garg N. J., Soman K. V., Zago M. P. (2016). Changes in proteome profile of peripheral blood mononuclear cells in chronic Chagas disease. *PLoS Neglected Tropical Diseases*.

[35] Tyagarajan K., Pretzer E. L., Wiktorowicz J. E. (2003). Thiol-reactive dyes for fluorescence labeling of proteomic samples. *Electrophoresis*.

[36] Pretzer E., Wiktorowicz J. E. (2008). Saturation fluorescence labeling of proteins for proteomic analyses. *Analytical Biochemistry*.

[37] Turck C. W., Falick A. M., Kowalak J. A. (2007). The Association of Biomolecular Resource Facilities Proteomics Research Group 2006 study: relative protein quantitation. *Molecular & Cellular Proteomics*.

[38] Nikolaienko R., Bovo E., Zima A. V. (2018). Redox dependent modifications of ryanodine receptor: basic mechanisms and implications in heart diseases. *Frontiers in Physiology*.

[39] Nakamura T., Lipton S. A. (2017). ‘SNO’-storms compromise protein activity and mitochondrial metabolism in neurodegenerative disorders. *Trends in Endocrinology and Metabolism*.

[40] Monteiro H. P., Costa P. E., Reis A. K., Stern A. (2015). Nitric oxide: protein tyrosine phosphorylation and protein s-nitrosylation in cancer. *Biomedical Journal*.

[41] Penna C., Sorge M., Femmino S., Pagliaro P., Brancaccio M. (2018). Redox aspects of chaperones in cardiac function. *Frontiers in Physiology*.

[42] Choi H., Tostes R. C., Webb R. C. (2011). S-nitrosylation inhibits protein kinase C-mediated contraction in mouse aorta. *Journal of Cardiovascular Pharmacology*.

[43] Yasukawa T., Tokunaga E., Ota H., Sugita H., Martyn J. A. J., Kaneki M. (2005). S-nitrosylation-dependent inactivation of Akt/protein kinase B in insulin resistance. *The Journal of Biological Chemistry*.

[44] Reynaert N. L., Ckless K., Korn S. H. (2004). Nitric oxide represses inhibitory *κ*B kinase through S-nitrosylation. *Proceedings of the National Academy of Sciences of the United States of America*.

[45] Coultrap S. J., Bayer K. U. (2014). Nitric oxide induces Ca^2+^-independent activity of the Ca^2+^/calmodulin-dependent protein kinase II (CaMKII). *The Journal of Biological Chemistry*.

[46] Hawkins H. L., Kramer A. F., Capaldi D. (1992). Aging, exercise, and attention. *Psychology and Aging*.

[47] Park H. S., Huh S. H., Kim M. S., Lee S. H., Choi E. J. (2000). Nitric oxide negatively regulates c-Jun N-terminal kinase/stress-activated protein kinase by means of S-nitrosylation. *Proceedings of the National Academy of Sciences of the United States of America*.

[48] Huang Z. M., Gao E., Fonseca F. V. (2013). Convergence of G protein-coupled receptor and S-nitrosylation signaling determines the outcome to cardiac ischemic injury. *Science Signaling*.

[49] Whalen E. J., Foster M. W., Matsumoto A. (2007). Regulation of *β*-adrenergic receptor signaling by S-nitrosylation of G-protein-coupled receptor kinase 2. *Cell*.

[50] Wang S.-B., Venkatraman V., Crowgey E. L. (2018). Protein *s*-nitrosylation controls glycogen synthase kinase 3*β* function independent of its phosphorylation state. *Circulation Research*.

[51] Hefetz-Sela S., Stein I., Klieger Y. (2014). Acquisition of an immunosuppressive protumorigenic macrophage phenotype depending on c-Jun phosphorylation. *Proceedings of the National Academy of Sciences of the United States of America*.

[52] Hernansanz-Agustin P., Izquierdo-Alvarez A., Garcia-Ortiz A., Ibiza S., Serrador J. M., Martinez-Ruiz A. (2013). Nitrosothiols in the immune system: signaling and protection. *Antioxidants & Redox Signaling*.

[53] Chang L., Goldman R. D. (2004). Intermediate filaments mediate cytoskeletal crosstalk. *Nature Reviews Molecular Cell Biology*.

[54] Mostowy S., Cossart P. (2012). Septins: the fourth component of the cytoskeleton. *Nature Reviews Molecular Cell Biology*.

[55] Huang Y. W., Yan M., Collins R. F., Diciccio J. E., Grinstein S., Trimble W. S. (2008). Mammalian septins are required for phagosome formation. *Molecular Biology of the Cell*.

[56] Park T., Chen Z. P., Leavitt J. (1994). Activation of the leukocyte plastin gene occurs in most human cancer cells. *Cancer Research*.

[57] Deady L. E., Todd E. M., Davis C. G. (2014). L-plastin is essential for alveolar macrophage production and control of pulmonary pneumococcal infection. *Infection and Immunity*.

[58] Todd E. M., Deady L. E., Morley S. C. (2013). Intrinsic T- and B-cell defects impair T-cell-dependent antibody responses in mice lacking the actin-bundling protein L-plastin. *European Journal of Immunology*.

[59] Seto S., Sugaya K., Tsujimura K., Nagata T., Horii T., Koide Y. (2013). Rab39a interacts with phosphatidylinositol 3-kinase and negatively regulates autophagy induced by lipopolysaccharide stimulation in macrophages. *PLoS One*.

[60] Zhang K., Zhang F., Yang J. M. (2018). Silencing of non-POU-domain-containing octamer-binding protein stabilizes atherosclerotic plaque in apolipoprotein E-knockout mice via NF-*κ*B signaling pathway. *International Journal of Cardiology*.

[61] Liepelt A., Mossanen J. C., Denecke B. (2014). Translation control of TAK1 mRNA by hnRNP K modulates LPS-induced macrophage activation. *RNA*.

