# Supplementary material for: Integrated Functional Analysis of the Nuclear Proteome of Classically and Alternatively Activated Macrophages
Source: Mediators Inflamm. 2019 Apr 30;2019:3481430. doi: 10.1155/2019/3481430 (PMC6515079; doi:10.1155/2019/3481430)
Supplement: Supplementary Materials — The bone-marrow derived murine macrophages were differentiated to proinflammatory (M1) and immunomodulatory (M2) phenotype by incubation with LPS/IFN-gamma and IL-4, respectively. Samples were incubated with (Asc+) or without (Asc−) ascorbate, resolved by the 2DE approach. Gels were labeled with BODIPY® FL N-(2-aminoethyl) maleimide and ProQ, images were analyzed with SameSpotst™ software, and normalized spot volumes were used for comparison. Protein spots with ≥|1.5|-fold change in abundance, S-nitrosylation level, or phosphorylation level (p < 0.05) in M1 or M2 macrophages (vs. control) were subjected to MALDI-TOF MS/MS analysis. pI: isoelectric pH; MW: molecular weight. [file 3481430.f1.pdf]

S1 Table: Integrated proteome profile of nuclear fractions of M1 and M2 macrophages [1]

Count	Protein name (Gene name)	Accession No.	Gel spot #	MW (kD)	pI	MS ID score	Abundance [2]			SNO (RoR) [3]			Phosphorylation [4]		
							M1v M0	M2v M0	M2v M1	M1v M0	M2v M0	M2v M1	M1v M0	M2v M0	M2v M1
1	14-3-3 protein gamma (YWHAG)	P61982	86	84	5.61	121	1.49	1.53	1.03	-2.29	-1.32	1.73	1.84	2.32	1.26
2	40S ribosomal protein (RPSA)	P14206	642	42	5.30	333	-1.13	-2.37	-2.10	2.02	1.95	-1.04	3.48	2.33	-1.49
3	60S ribosomal protein P2 (RPLP2)	P99027	54	97	5.94	65	-1.75	-1.02	1.72	-1.86	-2.93	-1.58	1.12	1.60	1.42
4	Actin, cytoplasmic 1 (ACTB)	P60710	118	72	6.07	313	-1.21	-1.50	-1.23	1.32	1.66	1.26	2.45	4.16	1.70
5	Actin, cytoplasmic 1 (ACTB)	P60710	612	12	8.02	203	1.58	1.08	-1.47	-1.49	-2.09	-1.41	2.05	1.87	-1.09
6	Actin, cytoplasmic 2 (ACTG1)	P63260	48	98	6.88	474	-2.09	-2.27	-1.09	-1.18	2.85	3.35	2.98	1.75	-1.70
7	Actin, gamma-smooth muscle (ACTG2)	P63268	668	16	4.51	259	-1.90	-3.54	-1.86	-1.05	1.33	1.39	-1.42	-3.40	-2.40
8	Actin, gamma smooth muscle (ACTG2)	P63268	713	49	8.33	257	-2.63	-1.66	1.59	1.04	-2.45	-2.56	100	1.28	-100
9	Apoptosis-inducing factor 2 (AIFM2)	Q8BUE4	896	37	6.80	32	-1.83	1.17	2.14	2.04	1.16	-1.77	2.82	-4.91	-13.84
10	Apoptosis-inducing factor 2 (AIFM2)	Q8BUE4	889	38	6.12	26	-1.13	1.13	1.28	1.60	1.86	1.16	1.41	2.15	1.52
11	Arf-GAP with SH3 domain, ANK repeat and PH domain protein 2 (ASAP2)	Q7SIG6	225	50	7.38	45	-1.70	-1.79	-1.06	1.04	-1.14	-1.18	6.40	4.09	-1.57
12	-do- ASAP2	Q7SIG6	563	17	4.38	36	-2.59	-2.21	1.17	1.12	1.10	-1.01	1.15	-1.63	-1.87
13	ATP-d- Clp protease subunit 3 (CLPP3)	Q9SXJ6	865	137	7.14	35	-4.64	1.34	6.23	2.24	-1.04	-2.34	-1.28	1.57	2.02
14	ATP-d- Clp protease CLPP3	Q9SXJ6	862	39	5.10	31	-1.66	1.03	1.70	-1.79	-1.13	1.59	1.35	3.24	2.40
15	Beta-galactoside alpha-2,6-sialyltransferase 1 (ST6GAL1)	Q64685	98	79	7.95	30	-1.37	1.23	1.69	-1.53	-1.04	1.47	-1.09	1.33	1.45
16	Cannabinoid receptor 2 (CNR2)	P47936	385	33	5.13	34	-1.53	-1.75	-1.14	1.56	-1.33	-2.07	1.42	-3.44	-4.87
17	CD59B glycoprotein (CD59B)	P58019	379	33	7.26	29	-6.92	-2.16	3.21	2.00	2.07	1.03	1.88	2.39	1.27
18	Cofilin-1 (CFL1)	P18760	151	65	5.28	72	1.55	-2.57	-4.00	-1.47	1.05	1.55	-1.01	1.07	1.08
19	Cytochrome P450 71B20 (CYP71B20)	Q9LTM3	828	65	7.45	28	-2.54	-1.52	1.67	-1.01	-1.10	-1.09	9.08	3.26	-2.79
20	Death ligand signal enhancer (KIAA0141)	Q9DCV6	354	35	7.06	48	-1.57	-1.14	1.38	-1.14	1.70	1.93	2.83	2.77	-1.02
21	Death ligand signal KIAA0141	Q9DCV6	503	22	7.83	42	2.46	-1.29	-3.18	-1.18	1.29	1.53	1.10	1.93	1.77
22	Death ligand signal KIAA0141	Q9DCV6	474	25	10.32	42	-2.24	-2.50	-1.11	6.93	2.51	-2.77	14.88	1.69	-8.82
23	Death ligand signal KIAA0141	Q9DCV6	392	32	10.69	42	-3.83	-2.26	1.69	2.81	-1.12	-3.14	3.14	-3.42	-10.75
24	Death ligand signal KIAA0141	Q9DCV6	335	36	6.05	42	-2.67	1.03	2.76	1.81	-1.03	-1.87	6.25	10.63	1.70
25	Death ligand signal KIAA0141	Q9DCV6	836	79	7.70	42	-1.75	1.35	2.37	-1.24	-1.01	1.23	1.37	1.73	1.26

26	Death ligand signal KIAA0141	Q9DCV6	928	88	5.29	42	-1.43	-1.93	-1.35	1.24	-1.05	-1.30	1.17	-1.68	-1.97
27	Death ligand signal KIAA0141	Q9DCV6	904	38	6.16	39	1.54	1.95	1.27	-1.15	-1.01	1.13	4.66	-100	-100
28	Death ligand signal KIAA0141	Q9DCV6	881	49	7.69	39	-2.80	-1.24	2.25	1.06	-1.17	-1.24	10.17	-1.82	-18.5
29	Death ligand signal KIAA0141	Q9DCV6	349	35	5.25	37	-1.08	-1.17	-1.08	1.04	-2.16	-2.25	2.00	1.75	-1.14
30	Death ligand signal KIAA0141	Q9DCV6	334	37	5.11	37	1.28	1.78	1.39	1.02	-1.19	-1.22	1.55	3.50	2.26
31	Death ligand signal KIAA0141	Q9DCV6	646	40	5.28	37	1.46	-1.45	-2.10	1.17	1.01	-1.16	100	1.28	-100
32	Death ligand signal KIAA0141	Q9DCV6	260	46	8.06	37	1.27	1.14	-1.11	1.12	4.27	3.81	1.10	-1.30	-1.43
33	Death ligand signal KIAA0141	Q9DCV6	67	92	4.99	36	-1.75	1.15	2.02	1.15	1.10	-1.04	1.05	1.29	1.23
34	Death ligand signal KIAA0141	Q9DCV6	479	25	6.22	35	-1.85	-1.10	1.69	1.20	2.12	1.77	1.50	-1.58	-2.37
35	Death ligand signal KIAA0141	Q9DCV6	367	34	4.66	35	-2.43	-2.33	1.05	-1.62	1.52	2.45	-4.39	-2.12	2.07
36	Death ligand signal KIAA0141	Q9DCV6	346	35	5.86	34	1.23	1.54	1.26	2.37	1.70	-1.39	4.65	-100	-100
37	Death ligand signal KIAA0141	Q9DCV6	229	50	7.04	34	-1.81	-2.68	-1.48	-1.08	2.68	2.88	100	100	2.12
38	Death ligand signal KIAA0141	Q9DCV6	898	26	8.00	33	-2.79	1.29	3.60	-1.10	-1.39	-1.26	17.69	-100	-100
39	Death ligand signal KIAA0141	Q9DCV6	384	33	5.10	33	1.25	-1.60	-2.01	-1.18	1.29	1.53	1.99	1.34	-1.48
40	Death ligand signal KIAA0141	Q9DCV6	645	39	5.29	33	1.46	-1.48	-2.16	1.36	1.71	1.26	5.08	1.96	-2.59
41	Death ligand signal KIAA0141	Q9DCV6	218	51	6.55	33	-2.13	-3.29	-1.55	1.26	1.28	1.02	3.96	5.99	1.51
42	Death ligand signal KIAA0141	Q9DCV6	509	22	6.84	32	2.58	1.11	-2.31	-1.11	1.19	1.32	6.22	-100	-100
43	Death ligand signal KIAA0141	Q9DCV6	328	37	4.59	32	-1.79	-3.24	-1.81	1.35	2.41	1.78	1.90	-1.55	-2.95
44	Death ligand signal KIAA0141	Q9DCV6	226	50	8.04	30	-1.73	-2.45	-1.42	-1.19	1.42	1.68	3.83	1.94	-1.97
45	Death ligand signal KIAA0141	Q9DCV6	163	63	7.60	30	-2.80	1.54	4.32	1.84	-1.27	-2.34	2.41	3.72	1.54
46	Death ligand signal KIAA0141	Q9DCV6	390	32	5.42	27	-1.40	-2.51	-1.80	-1.61	1.39	2.23	1.21	1.46	1.20
47	Death ligand signal KIAA0141	Q9DCV6	169	62	6.07	27	1.04	1.05	1.01	1.38	1.51	1.09	-1.17	1.35	1.58
48	DNA mismatch repair protein (MSH3)	P13705	127	72	6.88	40	-1.40	-1.09	1.29	-1.63	-1.19	1.37	2.89	5.81	2.01
49	Elongation factor 1-alpha 1 (EEF1A1)	P10126	910	76	6.21	68	-1.59	-1.10	1.44	-1.26	1.14	1.43	7.72	3.24	-2.38
50	Elongation factor 1-delta (EEF1D)	P57776	472	26	8.88	90	-1.41	1.96	2.77	-1.18	-1.50	-1.27	1.04	-100	-100
51	Enoyl-CoA hydratase, mitoch (ECHS1)	Q8BH95	480	25	6.47	70	-1.79	-1.37	1.31	-1.03	1.65	1.69	4.12	3.94	-1.05
52	Euk initiation factor 4A-I (EIF4A1)	P60843	359	34	6.64	134	-1.48	-1.32	1.12	-1.22	1.51	1.84	7.43	5.44	-1.37
53	Far upstream element-binding protein 1 (FUBP1)	Q91WJ8	332	37	6.66	44	-1.45	1.23	1.77	-1.05	-1.09	-1.04	2.48	1.67	-1.49
54	Far upstream element-binding protein 2 (KHSRP)	Q3U0V1	422	29	4.32	247	-1.87	-2.81	-1.50	-2.35	-1.15	2.04	100	100	-2.55
55	Fructose-bisphosphate aldolase A (ALDOA)	P05064	849	68	7.43	37	-2.23	1.41	3.13	1.16	-1.16	-1.34	1.33	2.24	1.68
56	Glycine-rich RNA-binding protein (RZ1A)	Q9LIN3	139	68	5.57	31	1.24	-1.87	-2.32	-1.39	-1.15	1.21	2.13	1.05	-2.03
57	GRB2-associated and regulator of MAPK protein-like (GAREML)	Q6PAJ3	168	62	6.03	50	-1.42	-1.40	1.01	2.17	2.43	1.12	-1.25	2.05	2.57

58	GRB2 regulator MAPK-like (GAREML)	Q6PAJ3	966	42	5.14	47	3.28	1.32	-2.49	-1.18	1.22	1.44	3.10	2.76	-1.12
59	GRB2 regulator MAPK-like (GAREML)	Q6PAJ3	298	41	7.20	43	2.75	2.03	-1.35	1.03	1.10	1.06	8.65	-3.27	-28.23
60	GRB2 regulator MAPK-like (GAREML)	Q6PAJ3	486	24	5.19	42	1.18	-1.01	-1.20	1.13	1.70	1.51	6.06	4.55	-1.33
61	GRB2 regulator MAPK-like (GAREML)	Q6PAJ3	415	30	5.27	42	-1.31	-1.60	-1.22	-1.25	1.43	1.78	-1.17	1.11	1.29
62	GRB2 regulator MAPK-like (GAREML)	Q6PAJ3	321	38	6.34	42	-1.36	-1.11	1.23	1.07	1.95	1.83	2.94	1.48	-1.99
63	GRB2 regulator MAPK-like (GAREML)	Q6PAJ3	452	27	5.03	41	1.40	-1.18	-1.65	-1.51	1.25	1.88	7.82	1.70	-4.59
64	GRB2 regulator MAPK-like (GAREML)	Q6PAJ3	467	26	7.19	40	-1.84	2.06	3.80	-1.51	1.00	1.51	3.61	-100	-100
65	GRB2 regulator MAPK-like (GAREML)	Q6PAJ3	286	44	5.39	37	-1.50	-1.96	-1.31	-1.21	-1.49	-1.23	4.30	3.07	-1.40
66	GRB2 regulator MAPK-like (GAREML)	Q6PAJ3	405	31	4.74	35	-2.88	-3.06	-1.06	-1.08	1.46	1.57	1.97	-57.9	-100
67	GRB2 regulator MAPK-like (GAREML)	Q6PAJ3	396	32	5.08	35	1.03	-1.05	-1.08	-1.09	-1.03	1.06	1.42	-1.13	-1.60
68	GRB2 regulator MAPK-like (GAREML)	Q6PAJ3	378	33	6.00	35	-1.16	1.62	1.89	1.77	-2.17	-3.85	1.71	1.30	-1.31
69	GRB2 regulator MAPK-like (GAREML)	Q6PAJ3	348	35	5.23	35	-1.09	-1.17	-1.07	-1.08	-1.87	-1.74	1.32	1.40	1.06
70	GRB2 regulator MAPK-like (GAREML)	Q6PAJ3	312	40	7.81	35	-1.55	1.31	2.04	-1.21	-1.04	1.17	2.07	1.79	-1.15
71	GRB2 regulator MAPK-like (GAREML)	Q6PAJ3	166	62	7.38	35	-1.24	1.32	1.65	1.13	1.03	-1.10	2.98	-1.24	-3.70
72	GRB2 regulator MAPK-like (GAREML)	Q6PAJ3	49	98	7.02	35	-2.09	-2.00	1.05	-1.19	2.09	2.49	3.90	2.33	-1.68
73	GRB2 regulator MAPK-like (GAREML)	Q6PAJ3	834	81	7.51	33	-2.50	-1.05	2.38	-1.13	-1.35	-1.19	-1.06	2.74	2.92
74	GRB2 regulator MAPK-like (GAREML)	Q6PAJ3	585	15	4.93	32	-3.27	1.13	3.71	1.43	-1.02	-1.46	1.69	-2.06	-3.48
75	GRB2 regulator MAPK-like (GAREML)	Q6PAJ3	315	39	7.72	30	-1.92	-1.21	1.59	-1.62	-1.21	1.35	1.51	-1.16	-1.76
76	Heat shock 71 kDa protein (HSPA8)	P63017	658	68	5.31	298	1.46	-1.47	-2.14	-1.02	-1.02	1.01	16.01	4.78	-3.35
77	Heat shock 71 kDa protein (HSPA8)	P63017	444	27	7.83	224	-4.86	1.19	5.78	-1.20	-1.79	-1.49	2.06	-100	-100
78	Heat shock 71 kDa protein (HSPA8)	P63017	522	20	5.21	175	1.52	2.04	1.34	-1.13	-1.30	-1.15	1.07	1.61	1.50
79	Heat shock 71 kDa protein (HSPA8)	P63017	660	68	5.28	174	1.27	-1.60	-2.03	1.17	1.16	-1.01	11.71	3.96	-2.95
80	Heat shock 71 kDa protein (HSPA8)	P63017	174	61	6.67	140	1.59	1.23	-1.29	1.07	1.73	1.62	4.23	4.30	1.02
81	Heterogeneous nuclear ribonucleoprotein A3 (HNRNPA3)	Q8BG05	908	48	6.16	331	-1.21	1.52	1.84	1.04	1.04	-1.00	15.30	-100	-100
82	Heterogeneous nuclear ribonucleoprotein F (HNRNPF)	Q9Z2X1	868	30	7.35	64	-1.94	-1.05	1.84	1.13	-1.57	-1.79	100	1.28	-100
83	Heterogeneous nuclear ribonucleoprotein K (HNRNPK)	P61979	995	59	6.46	141	1.41	1.71	1.21	1.02	1.07	1.05	1.58	1.15	-1.38
84	Heterogeneous nuclear ribonucleoprotein A2/B1 (HNRNPA2B1)	O88569	845	79	7.08	361	-2.06	-1.10	1.87	-1.10	-1.25	-1.13	2.97	5.65	1.90
85	Heterogeneous nuclear ribonucleoproteins C1/C2 (HNRNPC)	Q9Z204	749	137	6.38	86	-3.00	-1.44	2.08	1.06	1.32	1.24	1.17	2.50	2.14
86	Integrin beta-7 (ITGV+B7)	P26011	356	35	8.11	44	-1.44	1.13	1.63	-1.20	-1.10	1.09	11.56	7.59	-1.52
87	Keratin, type I cytoskeletal 10 (KRT10)	P02535	388	33	5.33	158	-2.15	-2.61	-1.21	-1.80	1.40	2.53	-1.52	1.42	2.17
88	Keratin, type I cytoskeletal 10 (KRT10)	P02535	57	97	5.99	65	-3.57	1.26	4.49	2.17	-2.10	-4.57	1.30	1.45	1.12
89	Keratin, type I cytoskeletal 10 (KRT10)	P02535	265	46	5.33	55	-2.35	1.15	2.70	1.11	1.07	-1.04	1.07	1.87	1.75

90	Keratin, type I cytoskeletal 10 (KRT10)	P02535	88	80	5.28	50	-1.60	-1.81	-1.13	-1.42	-1.12	1.26	-4.10	-1.24	3.29
91	KH domain protein At5g56140	Q9FKT4	656	67	5.34	32	1.37	-1.39	-1.90	-1.04	-1.12	-1.07	10.68	1.30	-8.24
92	L-type lectin-domain containing receptor kinase VIII.2 (LECRK82)	Q9LYX1	902	28	4.92	34	-1.15	-1.32	-1.15	-1.08	1.18	1.28	1.77	1.09	-1.62
93	Microtubule-actin cross-linking factor 1 (MACF1)	Q9QXZ0	846	75	7.12	37	-1.88	-1.21	1.56	1.06	1.14	1.08	7.54	7.90	1.05
94	Microtubule-actin cross-linking factor 1 (MACF1)	Q9QXZ0	876	137	6.00	32	-7.32	-1.42	5.15	2.69	-1.00	-2.70	3.51	4.05	1.15
95	Microtubule-actin cross-linking factor 1 (MACF1)	Q9QXZ0	419	30	5.47	29	1.03	1.19	1.16	-1.95	-1.20	1.63	1.63	-1.62	-2.64
96	Mitotic-spindle organizing protein 1A (GIP2)	Q9C9T3	554	17	6.02	30	1.48	2.16	1.45	1.08	-3.48	-3.75	100	100	-4.11
97	Nascent polypeptide-assoc complex subunit alpha, muscle-specific (NACA)	P70670	374	34	7.11	34	-2.45	-1.07	2.29	1.11	1.17	1.06	3.82	3.60	-1.06
98	Non-POU domain-containing octamer-binding protein (NONO)	Q99K48	917	88	5.28	137	-1.17	-1.76	-1.50	1.10	-1.13	-1.25	2.89	1.73	-1.67
99	Non-POU domain-containing octamer-binding protein (NONO)	Q99K48	911	80	6.25	118	-1.29	-2.40	-1.86	-1.21	1.77	2.14	2.75	3.33	1.21
100	Oxysterols receptor LXR-beta (NR1H2)	Q60644	851	64	5.50	32	-2.00	-1.96	1.02	1.85	-1.42	-2.64	1.02	2.59	2.53
101	Paraspeckle component 1 (PSPC1)	Q8R326	848	97	7.62	77	-1.75	1.04	1.82	1.32	-1.52	-2.01	1.13	1.40	1.24
102	Pentatricopeptide repeat-containing protein At1g77405	Q1PFC5	815	97	6.77	30	-2.18	-1.98	1.10	1.04	2.16	2.06	1.22	-1.11	-1.36
103	Pentatricopeptide repeat At2g15820	Q9XIL5	350	35	6.95	48	-2.08	1.07	2.23	1.03	1.02	-1.01	3.21	1.90	-1.69
104	Pentatricopeptide repeat At5g27270	O04647	122	72	7.24	40	-1.96	-1.06	1.85	-1.68	-1.15	1.46	1.00	-1.47	-1.47
105	Peroxidase 10 (PER10)	Q9FX85	992	33	9.24	42	-1.04	1.62	1.69	2.13	2.10	-1.01	100	1.28	-100
106	Peroxidase 10 (PER10)	Q9FX85	867	30	7.33	40	-1.90	-1.61	1.18	-1.05	-1.03	1.02	100	1.28	-100
107	Peroxidase 10 (PER10)	Q9FX85	850	65	7.37	40	-1.27	1.19	1.51	1.11	-1.44	-1.60	100	100	-2.80
108	Peroxidase 10 (PER10)	Q9FX85	831	78	7.43	38	-1.27	1.12	1.42	-1.72	-2.32	-1.35	3.88	1.21	-3.22
109	Peroxidase 10 (PER10)	Q9FX85	623	12	7.61	37	1.57	-1.19	-1.87	1.53	-1.10	-1.69	10.31	3.36	-3.07
110	Peroxidase 10 (PER10)	Q9FX85	678	46	9.49	36	1.69	1.56	-1.08	-1.03	-1.28	-1.24	100	1.28	-100
111	Peroxidase 10 (PER10)	Q9FX85	672	35	9.16	35	-1.10	1.39	1.53	1.58	1.47	-1.07	17.49	16.19	-1.08
112	Peroxidase 10 (PER10)	Q9FX85	895	55	8.22	35	-1.48	1.21	1.79	1.08	-2.99	-3.23	5.02	2.52	-1.99
113	Peroxidase 10 (PER10)	Q9FX85	760	144	6.77	35	-2.58	1.49	3.85	1.83	-1.29	-2.36	-1.14	3.43	3.90
114	Peroxidase 10 (PER10)	Q9FX85	576	16	6.32	32	-1.03	1.84	1.91	1.82	-1.37	-2.49	3.52	-1.52	-5.35
115	Peroxidase 10 (PER10)	Q9FX85	943	60	5.41	32	-1.61	-1.03	1.55	1.31	-1.16	-1.52	-1.20	1.06	1.27
116	Peroxiredoxin-1 (PRDX1)	P35700	686	27	4.88	73	-1.83	-1.84	-1.00	2.01	2.01	-1.00	3.30	-1.55	-5.12
117	Phospholipase D zeta (PLDZETA)	P58766	667	16	4.43	35	-2.94	-3.63	-1.23	1.03	-1.04	-1.08	-2.41	-4.38	-1.82
118	Phospholipase D zeta (PLDZETA)1	P58766	770	125	7.18	35	-3.12	-1.02	3.07	-1.34	-1.92	-1.43	3.16	-2.63	-8.31
119	Phospholipase D zeta (PLDZETA)	P58766	856	66	7.75	31	-1.71	-1.06	1.61	-1.03	-1.48	-1.43	6.46	2.54	-2.54
120	Phospholipase D zeta (PLDZETA)	P58766	892	97	7.00	30	-2.09	-1.39	1.50	-1.26	2.33	2.94	1.31	1.98	1.51

121	Plastin-2 (LCP1)	Q61233	571	16	5.00	167	-1.78	-2.40	-1.35	-1.69	1.63	2.76	3.95	1.38	-2.88
122	Probable ATP-d- RNA helicase (DDX5)	Q61656	621	12	5.29	85	1.75	1.48	-1.18	-1.86	-2.36	-1.27	100	100	-38.15
123	Prohibitin (PHB)	P67778	142	68	5.40	151	-1.43	-2.16	-1.51	1.19	-1.09	-1.29	6.93	2.56	-2.71
124	Protein Hook homolog (HOOK1)	Q8BIL5	528	19	4.37	29	3.95	1.94	-2.04	-1.31	1.12	1.47	3.05	-1.61	-4.90
125	Protein kinase C and casein kinase substrate in neurons protein 1 (PACSIN1)	Q61644	209	54	6.96	39	-1.10	-1.03	1.07	-1.18	-1.61	-1.36	2.62	3.45	1.32
126	Protein kinase C and casein kinase substrate in neurons protein 1 (PACSIN1)	Q61644	194	57	6.85	39	2.25	1.80	-1.25	-1.50	1.14	1.70	2.01	-1.23	-2.48
127	Protein kinase C and casein kinase substrate in neurons protein 1 (PACSIN1)	Q61644	211	53	7.15	34	1.50	1.56	1.04	-1.17	-1.97	-1.68	1.63	2.35	1.44
128	Protein kinase C and casein kinase substrate in neurons protein 1 (PACSIN1)	Q61644	212	53	7.53	34	2.41	1.26	-1.92	-3.13	-3.31	-1.06	3.59	2.33	-1.54
129	Protein kinase C and casein kinase substrate in neurons protein 1 (PACSIN1)	Q61644	115	73	7.02	34	-2.21	-1.18	1.87	1.31	1.43	1.09	3.24	-1.05	-3.40
130	Protein kinase C and casein kinase substrate in neurons protein 1 (PACSIN1)	Q61644	82	86	10.49	34	-1.49	-1.90	-1.27	3.58	3.33	-1.08	1.69	1.20	-1.40
131	Protein kinase C and casein kinase substrate in neurons protein 1 (PACSIN1)	Q61644	175	61	6.83	32	1.90	1.56	-1.22	1.14	1.78	1.56	3.97	1.46	-2.72
132	Protein kinase C and casein kinase substrate in neurons protein 1 (PACSIN1)	Q61644	78	89	5.99	32	-1.76	1.13	1.98	1.45	-1.61	-2.34	-1.02	2.62	2.66
133	Protein kinase C and casein kinase substrate in neurons protein 1 (PACSIN1)	Q61644	126	72	6.78	30	-2.78	-1.25	2.23	1.38	1.17	-1.17	2.74	2.29	-1.19
134	Putative F-box protein At1g31090	Q9SA03	164	62	6.18	39	1.55	1.29	-1.21	-1.00	1.15	1.16	1.03	1.95	1.90
135	Putative F-box protein At1g31090	Q9SA03	502	22	5.07	37	1.88	1.06	-1.77	-1.34	1.23	1.65	6.30	2.18	-2.89
136	Putative F-box protein At1g31090	Q9SA03	162	63	6.29	37	1.54	1.01	-1.52	-1.09	1.25	1.36	1.35	2.60	1.93
137	Putative F-box protein At1g31090	Q9SA03	505	22	9.08	33	10.33	1.51	-6.84	-7.88	-1.37	5.73	2.61	1.18	-2.22
138	Putative F-box protein At1g31090	Q9SA03	743	94	9.55	33	2.86	1.54	-1.86	-4.30	-1.31	3.29	1.26	2.02	1.61
139	Putative F-box protein At1g31090	Q9SA03	588	15	4.74	28	1.44	-2.25	-3.23	-3.08	1.38	4.27	1.19	-6.19	-7.36
140	Ras-related protein (RAB39A)	Q8BHD0	270	46	7.20	51	1.48	1.21	-1.23	-1.27	1.56	1.99	5.96	4.61	-1.29
141	Ras-related protein (RAB39A)	Q8BHD0	368	34	5.64	45	-2.59	-5.52	-2.13	-1.39	2.98	4.15	1.17	-1.16	-1.36
142	Ras-related protein (RAB39A)	Q8BHD0	564	17	6.22	40	-2.44	-1.06	2.31	2.40	1.74	-1.38	1.03	-1.55	-1.61
143	Ras-related protein (RAB39A)	Q8BHD0	534	18	4.99	40	1.57	-1.46	-2.28	1.44	3.56	2.46	10.79	5.51	-1.96
144	Ras-related protein (RAB39A)	Q8BHD0	536	18	7.36	40	-2.57	-1.52	1.69	-1.52	-1.38	1.10	10.83	11.51	1.06
145	Ras-related protein (RAB39A)	Q8BHD0	460	26	8.77	40	-2.08	1.34	2.80	1.39	1.09	-1.27	2.35	-2.13	-5.00
146	Ras-related protein (RAB39A)	Q8BHD0	463	26	10.73	40	-2.62	-2.37	1.11	2.75	1.08	-2.56	-1.41	-3.28	-2.32
147	Ras-related protein (RAB39A)	Q8BHD0	447	27	5.33	40	-2.37	-2.35	1.01	-1.06	1.23	1.30	66.58	14.44	-4.61
148	Ras-related protein (RAB39A)	Q8BHD0	438	28	7.28	40	-2.00	1.62	3.25	-1.18	-1.59	-1.34	8.06	-1.19	-9.58
149	Ras-related protein (RAB39A)	Q8BHD0	439	28	6.88	40	-1.06	1.76	1.87	1.18	1.57	1.33	58.74	-100	-100
150	Ras-related protein (RAB39A)	Q8BHD0	430	29	5.39	40	-2.63	-1.08	2.43	1.22	1.15	-1.06	4.28	2.92	-1.47

151	Ras-related protein (RAB39A)	Q8BHD0	420	30	5.53	40	-1.86	-1.01	1.84	-1.83	-1.57	1.17	2.14	1.56	-1.37
152	Ras-related protein (RAB39A)	Q8BHD0	369	34	5.51	40	-1.35	-2.15	-1.59	-1.93	1.35	2.61	-1.02	-1.52	-1.49
153	Ras-related protein (RAB39A)	Q8BHD0	372	34	5.42	40	-1.02	-1.77	-1.73	-1.72	1.37	2.36	1.57	-1.40	-2.19
154	Ras-related protein (RAB39A)	Q8BHD0	355	35	7.62	40	-1.21	1.05	1.27	-1.12	1.15	1.30	3.48	3.51	1.01
155	Ras-related protein (RAB39A)	Q8BHD0	297	41	6.45	40	2.42	1.62	-1.49	1.20	1.30	1.09	5.29	2.13	-2.49
156	Ras-related protein (RAB39A)	Q8BHD0	967	43	5.07	40	1.70	-1.57	-2.67	1.68	2.23	1.33	3.36	1.33	-2.52
157	Ras-related protein (RAB39A)	Q8BHD0	786	51	6.84	38	2.04	1.49	-1.37	-1.46	-2.70	-1.85	3.18	1.69	-1.88
158	Ras-related protein (RAB39A)	Q8BHD0	562	17	6.85	36	-2.47	-1.11	2.24	1.74	1.67	-1.04	4.64	1.11	-4.20
159	Ras-related protein (RAB39A)	Q8BHD0	416	30	5.33	36	-1.14	-1.65	-1.45	-1.88	1.16	2.18	1.75	1.20	-1.46
160	Ras-related protein (RAB39A)	Q8BHD0	376	33	6.88	36	-2.34	1.21	2.84	-1.05	-1.24	-1.18	2.84	5.82	2.05
161	Ras-related protein (RAB39A)	Q8BHD0	296	42	6.88	36	1.99	1.65	-1.21	-1.28	1.48	1.89	3.18	2.61	-1.22
162	Ras-related protein (RAB39A)	Q8BHD0	234	49	7.50	36	-3.40	-1.98	1.71	-1.13	1.18	1.33	100	100	-36.4
163	Ras-related protein (RAB39A)	Q8BHD0	785	52	6.78	36	-1.78	-1.63	1.09	1.05	-1.14	-1.20	100	100	-2.74
164	Ras-related protein (RAB39A)	Q8BHD0	153	65	7.97	36	-1.85	2.29	4.24	1.38	-1.60	-2.20	4.14	4.40	1.06
165	Ras-related protein (RAB39A)	Q8BHD0	143	67	5.37	36	1.13	-1.59	-1.80	1.03	-1.08	-1.11	8.12	2.37	-3.43
166	Ras-related protein (RAB39A)	Q8BHD0	140	68	5.45	36	-2.11	-3.71	-1.75	1.03	1.18	1.15	1.69	1.24	-1.36
167	Ras-related protein (RAB39A)	Q8BHD0	39	106	10.63	36	-2.06	-1.02	2.02	1.92	1.04	-1.85	-2.04	1.09	2.22
168	Ras-related protein (RAB39A)	Q8BHD0	752	137	6.20	36	-4.20	-1.34	3.13	1.03	-1.46	-1.51	1.03	1.91	1.86
169	Ras-related protein (RAB39A)	Q8BHD0	773	137	7.61	36	-2.26	2.21	4.98	1.51	-1.63	-2.47	1.11	1.42	1.27
170	Ras-related protein (RAB39A)	Q8BHD0	18	138	8.03	36	1.11	1.48	1.33	-1.82	1.22	2.21	1.49	1.59	1.07
171	Ras-related protein (RAB39A)	Q8BHD0	138	68	5.50	34	-1.39	-2.53	-1.81	-1.55	-1.12	1.38	1.85	-1.13	-2.09
172	Ras-related protein (RAB39A)	Q8BHD0	532	18	5.46	33	1.11	-1.07	-1.19	-2.43	1.33	3.23	-1.92	-6.33	-3.29
173	Ras-related protein (RAB39A)	Q8BHD0	718	27	4.92	33	-1.05	-1.42	-1.36	1.14	1.13	-1.01	3.84	1.14	-3.37
174	Ras-related protein (RAB39A)	Q8BHD0	740	95	9.98	32	-1.02	1.09	1.10	1.69	1.21	-1.39	1.01	1.32	1.31
175	Ras-related protein (RAB39A)	Q8BHD0	533	18	5.05	31	4.09	1.20	-3.40	-1.42	1.60	2.27	8.02	2.45	-3.27
176	Ras-related protein (RAB39A)	Q8BHD0	643	40	5.26	30	1.59	-1.37	-2.19	1.16	1.43	1.23	66.42	14.60	-4.55
177	Ras-related protein (RAB39A)	Q8BHD0	336	36	6.28	29	-1.69	1.36	2.29	-1.02	1.10	1.13	3.20	3.94	1.23
178	Septin-2 (SEPT2)	P42208	617	12	8.27	90	2.72	1.40	-1.95	-1.92	-2.72	-1.41	1.65	-2.50	-4.12
179	Spermidine hydroxycinnamoyl transferase (SHT)	O64470	809	194	7.39	33	-1.08	1.74	1.89	-2.25	-4.70	-2.09	2.92	1.28	-2.29
180	Splicing factor, proline- and glutamine-rich (SFPQ)	Q8VIJ6	930	97	5.47	266	2.01	1.34	-1.50	-1.52	1.24	1.89	-1.02	1.61	1.64
181	Splicing factor, proline- and glutamine-rich (SFPQ)	Q8VIJ6	319	39	6.86	175	-1.33	-1.04	1.28	-1.15	1.54	1.77	1.67	1.94	1.16
182	Splicing factor, proline- and glutamine-rich (SFPQ)	Q8VIJ6	537	18	10.6	32	-1.30	-2.46	-1.89	3.00	2.95	-1.02	1.74	-6.21	-10.8

183	Stress-70 protein, mitochondrial (HSPA9)	P38647	171	62	6.33	460	1.85	1.23	-1.50	1.17	1.36	1.16	1.52	2.66	1.75
184	Succinate dehydrogenase assembly factor 1, mitochondrial (SDHAF1)	Q3U276	669	15	4.61	42	-3.44	-6.57	-1.91	-1.14	2.02	2.30	-3.74	-5.23	-1.40
185	Transcription cofactor HES-6 (HES6)	Q9JHE6	520	20	5.43	49	-1.37	-1.80	-1.32	-1.16	1.27	1.47	21.70	19.22	-1.13
186	Transcription cofactor HES-6 (HES6)	Q9JHE6	446	27	4.73	48	-1.55	-3.32	-2.15	-1.07	1.48	1.58	1.49	-2.84	-4.21
187	Transcription cofactor HES-6 (HES6)	Q9JHE6	727	97	5.50	47	-1.02	1.04	1.06	-2.40	-1.04	2.30	1.03	2.60	2.53
188	Transcription cofactor HES-6 (HES6)	Q9JHE6	145	67	8.24	46	-1.72	-1.05	1.63	1.29	-1.65	-2.13	1.87	3.38	1.81
189	Transcription cofactor HES-6 (HES6)	Q9JHE6	497	23	5.49	44	1.63	-1.59	-2.59	-1.07	1.67	1.79	4.68	4.92	1.05
190	Transcription cofactor HES-6 (HES6)	Q9JHE6	873	66	7.79	44	-2.64	-1.31	2.02	-1.41	-1.37	1.03	4.08	1.71	-2.39
191	Transcription cofactor HES-6 (HES6)	Q9JHE6	499	23	5.19	43	3.14	1.23	-2.54	1.30	1.97	1.51	4.42	2.09	-2.12
192	Transcription cofactor HES-6 (HES6)	Q9JHE6	435	28	4.72	43	-1.47	-2.48	-1.68	-1.05	1.61	1.70	1.48	-1.16	-1.72
193	Transcription cofactor HES-6 (HES6)	Q9JHE6	432	29	7.02	43	-4.17	-2.07	2.01	-1.09	1.17	1.28	4.26	3.21	-1.33
194	Transcription cofactor HES-6 (HES6)	Q9JHE6	698	58	9.60	43	1.72	1.47	-1.17	-1.32	1.17	1.54	100	1.28	-100
195	Transcription cofactor HES-6 (HES6)	Q9JHE6	905	37	6.22	42	-1.95	-1.25	1.56	1.08	3.01	2.79	3.77	5.35	1.42
196	Transcription cofactor HES-6 (HES6)	Q9JHE6	789	125	7.39	42	-3.45	-1.11	3.10	1.00	-1.38	-1.39	11.84	3.78	-3.14
197	Transcription cofactor HES-6 (HES6)	Q9JHE6	360	34	6.75	41	-1.11	1.54	1.71	-1.03	1.11	1.15	3.94	1.43	-2.75
198	Transcription cofactor HES-6 (HES6)	Q9JHE6	568	17	5.19	40	-2.18	-1.19	1.83	1.41	1.04	-1.35	100	1.28	-100
199	Transcription cofactor HES-6 (HES6)	Q9JHE6	526	19	6.47	40	1.21	-1.94	-2.35	-1.01	2.28	2.31	4.89	-1.42	-6.97
200	Transcription cofactor HES-6 (HES6)	Q9JHE6	722	35	8.61	40	1.44	2.19	1.52	-2.20	-1.20	1.83	8.53	-100	-100
201	Transcription cofactor HES-6 (HES6)	Q9JHE6	855	68	7.72	39	-2.32	1.41	3.28	-1.09	-1.44	-1.33	4.23	1.16	-3.65
202	Transcription cofactor HES-6 (HES6)	Q9JHE6	970	73	10.0	39	-1.22	1.20	1.47	1.20	1.01	-1.19	3.00	2.25	-1.33
203	Transcription cofactor HES-6 (HES6)	Q9JHE6	484	25	6.97	38	-1.52	1.05	1.60	-1.98	1.39	2.74	4.62	-3.41	-15.7
204	Transcription cofactor HES-6 (HES6)	Q9JHE6	694	65	9.47	38	-1.14	-1.50	-1.31	-1.70	-1.10	1.55	1.04	-1.04	-1.07
205	Transcription cofactor HES-6 (HES6)	Q9JHE6	762	127	6.78	38	-1.26	2.09	2.63	-2.13	-1.79	1.19	2.34	5.45	2.33
206	Transcription cofactor HES-6 (HES6)	Q9JHE6	478	25	5.55	37	-1.67	1.04	1.75	-1.97	-1.59	1.24	-1.07	1.47	1.56
207	Transcription cofactor HES-6 (HES6)	Q9JHE6	854	36	7.00	37	-1.48	1.24	1.84	1.01	-1.36	-1.36	2.83	-1.76	-4.99
208	Transcription cofactor HES-6 (HES6)	Q9JHE6	969	75	9.82	37	-1.35	-1.05	1.28	2.20	1.49	-1.47	2.54	2.29	-1.11
209	Transcription cofactor HES-6 (HES6)	Q9JHE6	586	15	5.00	36	1.07	1.31	1.22	-2.60	1.02	2.66	7.97	1.00	-7.94
210	Transcription cofactor HES-6 (HES6)	Q9JHE6	551	18	7.86	36	-1.67	-2.61	-1.56	-1.07	-1.42	-1.33	26.14	9.36	-2.79
211	Transcription cofactor HES-6 (HES6)	Q9JHE6	774	125	7.62	36	-3.16	1.00	3.17	-1.07	-1.05	1.02	1.63	-6.81	-11.1
212	Transcription cofactor HES-6 (HES6)	Q9JHE6	590	15	4.85	35	3.67	3.24	-1.13	1.06	-1.48	-1.57	2.17	1.35	-1.61
213	Transcription cofactor HES-6 (HES6)	Q9JHE6	879	50	7.73	35	-1.16	-2.10	-1.82	-1.05	-1.05	1.01	100	100	-6.10
214	Transcription cofactor HES-6 (HES6)	Q9JHE6	700	58	9.36	35	1.82	2.28	1.25	-1.41	-1.77	-1.26	4.99	-100	-100
215	Transcription cofactor HES-6 (HES6)	Q9JHE6	779	137	7.87	35	-2.07	1.29	2.66	1.55	1.29	-1.20	2.01	-2.76	-5.55

216	Transcription cofactor HES-6 (HES6)	Q9JHE6	599	14	8.12	34	1.48	-1.50	-2.22	1.22	1.13	-1.07	2.03	-1.58	-3.22
217	Transcription cofactor HES-6 (HES6)	Q9JHE6	421	30	5.65	34	-2.96	-1.57	1.89	2.49	2.91	1.17	10.69	5.29	-2.02
218	Transcription cofactor HES-6 (HES6)	Q9JHE6	853	37	6.95	34	-3.24	-1.27	2.54	-1.39	2.04	2.83	32.58	23.76	-1.37
219	Transcription cofactor HES-6 (HES6)	Q9JHE6	295	42	5.77	34	1.02	-1.01	-1.03	1.56	-1.38	-2.16	2.20	2.66	1.21
220	Transcription cofactor HES-6 (HES6)	Q9JHE6	714	49	8.36	34	-2.31	-1.77	1.31	-1.01	-1.44	-1.43	100	100	-20.1
221	Transcription cofactor HES-6 (HES6)	Q9JHE6	650	39	5.04	33	-3.31	-2.09	1.58	1.07	1.11	1.03	1.48	1.17	-1.26
222	Transcription cofactor HES-6 (HES6)	Q9JHE6	877	50	7.62	32	-2.03	-2.09	-1.03	-1.55	1.03	1.60	8.81	2.54	-3.47
223	Transcription cofactor HES-6 (HES6)	Q9JHE6	670	15	4.67	30	-3.22	-4.67	-1.45	1.02	1.47	1.45	-1.64	-6.20	-3.78
224	Transcription cofactor HES-6 (HES6)	Q9JHE6	958	37	6.53	30	-1.41	-1.11	1.27	-1.07	1.86	1.99	100	1.28	-100
225	Transcription cofactor HES-6 (HES6)	Q9JHE6	894	58	8.18	30	-1.41	1.07	1.51	-1.95	-1.46	1.34	4.53	-1.26	-5.69
226	Transcription cofactor HES-6 (HES6)	Q9JHE6	436	28	4.42	29	-1.21	-3.01	-2.50	-1.61	1.97	3.17	34.74	9.51	-3.65
227	Transcription cofactor HES-6 (HES6)	Q9JHE6	942	60	5.43	29	-1.81	-1.20	1.50	1.78	-1.05	-1.88	-1.71	1.29	2.21
228	Transcription cofactor HES-6 (HES6)	Q9JHE6	852	60	5.51	27	-1.99	-1.73	1.16	1.32	-1.38	-1.81	1.19	2.01	1.68
229	Transcription initiation factor TFIID subunit 14 (TAF14)	F4IPK2	443	27	5.44	37	-1.97	-1.79	1.10	-1.07	1.33	1.42	5.58	1.62	-3.45
						Increase	**37**	**34**	**89**	**36**	**50**	**66**	**164**	**118**	**40**
						Decrease	**108**	**71**	**50**	**42**	**31**	**38**	**9**	**46**	**122**

[1] The nuclear protein samples from M1, M2 and M0 macrophages (n=4 per group) were incubated with (Asc+) or without (Asc-) ascorbate and resolved by 2D-GE approach. Gels were labeled with BODIPY FL N- (2-aminoethyl) maleimide, and gels were imaged for BD fluorescence and were analyzed with SameSpots software. Next, the gels were scanned to calculate BD fluorescence spill-over in Pro-Q Diamond fluorescence detection range, and then labeled with Pro-Q Diamond and imaged for phosphorylation profile. The normalized spot volumes were used for comparison. Proteins spots with ≥ |1.5| fold change in abundance, S-nitrosylation level or phosphorylation level ($p<0.05$) in M1 or M2 macrophages (vs. M0 controls) were subjected to MALDI-TOF MS/MS analysis and those identified with high (score >60) or mid-level (>40) confidence are highlighted in light brown and light blue colors, respectively.

[2] Ratiometric calculation from BODIPY-fluorescence units in Asc^+ aliquots (normal vs experimental) was conducted for quantifying the differential abundance of protein spots (Δ protein abundance = Asc^+ M1 or M2 / Asc^+ M0).

[3] The ratio of ratios, i.e. RoR= [Asc^- M1 or M2 / Asc^- M0] / [Asc^+ M1 or M2 / Asc^+ M0] was calculated to obtain the change in S-NO levels normalized for protein abundance. As S-NO modification inhibits the Cys-BODIPY fluorescence; a negative RoR value would indicate an increase in S-NO levels (and vice versa).

[4] Ratiometric calculation from Pro-Q Diamond fluorescence units (after subtracting BD spill-over) in normal vs experimental gels was conducted for quantifying the differential phosphorylation of protein spots (Δ protein phosphorylation = M1 or M2 PQD / M0 PQD).

The dark/light green and dark/light pinkish/brown colors indicate an increase and decrease in abundance, RoR values, and phosphorylation levels, respectively, in M1 and M2 (vs. M0) macrophages. The darker shade means the higher value.
